# Physical Activity and Risks of Cardiovascular Diseases: A Mendelian Randomization Study

**DOI:** 10.3389/fcvm.2021.722154

**Published:** 2021-09-29

**Authors:** Chengui Zhuo, Jianqiang Zhao, Miao Chen, Yunlong Lu

**Affiliations:** ^1^Department of Cardiology, Taizhou Central Hospital (Taizhou University Hospital), Taizhou, China; ^2^Department of Cardiology, The Fourth Affiliated Hospital of Zhejiang University School of Medicine, Zhejiang, China; ^3^Department of Cardiology and Atrial Fibrillation Center, The First Affiliated Hospital, College of Medicine, Zhejiang University, Hangzhou, China

**Keywords:** physical activity, cardiovascular disease, coronary artery disease, myocardial infarction, mendelian randomization

## Abstract

**Background:** Although some observational studies have shown that physical activity may have a positive relationship with cardiovascular diseases, the causal effect remains uncertain. We conducted a Mendelian randomization (MR) study to identify the potential causal effect between physical activity and cardiovascular diseases.

**Methods:** Summary statistics of genome-wide association studies on four physical activity phenotypes and cardiovascular diseases were utilized. MR analysis was performed using inverse-variance weighted (IVW) and multivariable MR. Multiple sensitivity analysis was further conducted to identify the robustness of our results.

**Results:** Genetically predicted self-reported vigorous physical activity (VPA) was significantly associated with lower risk of myocardial infarction (IVW OR: 0.24, 95% CI: 0.08–0.68, *p*-value: 0.007). Additionally, the causal effect of VPA with myocardial infarction was robust after adjusting for several cardiovascular risk factors through using the multivariable MR. There were no apparent causal associations between physical activity with other cardiovascular diseases. Results were consistent with the sensitivity analysis.

**Conclusion:** The present study supports a protective role of self-reported vigorous physical activity in the initiation of myocardial infarction and highlights the importance of activity levels of physical activity. Further studies are required to elucidate the potential biological pathways of physical activity with cardiovascular diseases.

## Background

Cardiovascular diseases (CVDs) represent a leading cause of death worldwide and, despite coordinated preventive efforts, still account for 30% of all global deaths and impose a substantial burden on society (1). From previous epidemiological studies, we have learned that lower systolic blood pressure (2), weight reduction (3), reduction of smoking (4), education (5), and healthy diet (6) would all lower the risk of CVDs. However, there are many other ideal preventive measures that need to be considered.

The association between physical activity and CVDs has attracted much attention in recent years. For example, previous studies have demonstrated that physical activity improves the prognosis of CVDs in both healthy participants and those with traditional risk factors (7–9). The 2016 European Guidelines on CVD prevention in clinical practice recommended that every person accumulate at least 150 min/week of moderate-intensity physical activity or 75 min/week of vigorous-intensity physical activity (10). Nevertheless, most of the evidence for these guidelines originates from observational studies, which cannot be used to identify the causality because of the possibility of confounding and reverse causation.

With regard to the causal relationship, several randomized controlled trials (RCTs) have been applied to show an inverse relationship between physical activity and overall CVD risk (11, 12). However, other RCTs found no such benefits (13, 14). The results of RCTs are still controversial due to the limited sample size and follow-up time. More critically, studies to date rely mostly on self-reported information, which might be influenced by memory inaccuracy and individuals' mood (7). Therefore, the causal associations of physical activity with CVDs remain uncertain.

Mendelian randomization (MR) is an alternative epidemiological approach that can use genetic variations as instrumental variables (IVs) to uncover the causal relationship between exposure and disease outcomes, and avoiding potential confounders in observational studies (15). MR has been previously used to explore the association of several risk factors and CVDs, such as smoking (16), body mass index (BMI) (17), and alcohol consumption (18). In this study, a two-sample MR study was performed to examine the potential causal association between physical activity and risk of CVDs using genetic variations associated with self-reported and objective accelerometer-based physical activity identified from published genome-wide association studies (GWASs).

## Methods

### Study Design

MR studies are widely adopted to investigate the association between exposure and outcomes. There are three key assumptions for MR: First, the genetic variants should be associated with the physical activity. Second, the genetic variants should not be associated with any confounders. Third, the genetic variants exert effects on the outcome only *via* the physical activity (Supplementary Figure 1). All summary-level data used in this study come from published GWASs on physical activity and CVDs, including coronary artery disease (CAD), myocardial infarction (MI), heart failure (HF), atrial fibrillation (AF), ischemic stroke (IS), and its subtypes.

### Data on PA

From the available literature, we note that there is a significant genetic influence on physical activity levels (19, 20). Specifically, previous studies have applied both human and mouse models, and the amount of heritability observed has ranged from 0.20 to 0.92. Genetic factors influence activity levels through aerodynamic performance, muscle strength, muscle endurance, and anaerobic performance (21). We used summary-level data from a recently published GWAS on physical activity conducted in participants from the UK Biobank study (22). This GWAS examined four physical activity phenotypes (Supplementary Figure 2) including self-reported moderate-to-vigorous physical activity (MVPA), self-reported vigorous physical activity (VPA), overall acceleration average, and fraction of accelerations > 425 milli-gravities (corresponding to an equivalent level of vigorous physical activity). In the UK Biobank, self-reported physical activity of 377,234 participants during work and leisure time was ascertained through a touchscreen questionnaire that was similar to the International Physical Activity Questionnaire (23). For the measurement of accelerometer-based physical activity, approximately 91,000 participants wore an Axivity-AX3 triaxial accelerometer on their wrist for 7 days (24). The cutoff value of 425 mg was chosen because it corresponds to an equivalent level of vigorous physical activity.

Genome-wide significant (*p*-value < 5 × 10^−8^) and independent (linkage disequilibrium *r*^2^ < 0.1) single-nucleotide polymorphisms (SNPs) were selected as instruments for the MR analysis. Finally, the GWAS identified nine and five independent genome-wide significant SNPs for MVPA and VPA, respectively. Furthermore, we also selected eight SNPs significantly associated with “overall acceleration average” and two SNPs associated with “fraction of accelerations > 425 milli-gravities (mg).” Given only two SNPs were available for “fraction of accelerations > 425 mg,” a more relaxed significance threshold (*p*-value < 5 × 10^−7^) was used and finally eight SNPs were detected. This method of relaxing the threshold, which is also called suggestive significance level, has been used in several previous MR studies when few significant SNPs are available (25, 26). When SNPs for the physical activity phenotypes were not available in the summary statistics of outcome GWAS, proxies (linkage disequilibrium *r*^2^ > 0.8) were identified *via* an online tool, available at: https://ldlink.nci.nih.gov/. Resulting lists of SNPs for each phenotype are given in Supplementary Table 1.

### Data on CVDs

Summary statistics for the associations of the physical activity-related SNPs with CVDs were extracted from the Coronary Artery Disease Genome-Wide Replication and Meta-analysis plus the Coronary Artery Disease Genetics (CardiogramplusC4D) consortium for CAD (60,801 cases and 123,504 controls) and MI (43,676 cases and 128,197 controls) (27), from the Heart Failure Molecular Epidemiology for Therapeutic Targets (HERMES) Consortium for heart failure (47,309 cases and 930, 014 controls) (28), from the Atrial Fibrillation Haplotype Reference Consortium for atrial fibrillation (65,446 cases and 522,744 controls) (29), and from the MEGASTROKE consortium for ischemic stroke and its subtypes (34,217 cases and 404,630 controls) (30). The definition of ischemic stroke was according to the Trial of Org 10172 in Acute Stroke Treatment criteria (31), and its subtypes were categorized as large artery stroke (4,373 cases and 146,392 controls), small vessel stroke cases (5,386 cases and 192,662 controls), and cardioembolic stroke cases (7,193 cases and 204,570 controls).

### Statistical Power

The *a priori* statistical power was calculated using a web-based application (http://cnsgenomics.com/shiny/mRnd/) (32). We identified that the nine SNPs for MVPA and the five SNPs for VPA explained 0.1% of the phenotypic variability. The eight SNPs for “overall acceleration average” and “fraction of accelerations > 425 mg” explained 0.3% of the phenotypic variability. Given a type 1 error of 5%, the power estimates of the four physical activity phenotypes are shown in Supplementary Table 2.

### Statistical Analysis

A two-sample MR method was used in the present study. We calculated the MR estimates of the effect of physical activity on CVDs through utilizing the Wald estimator. The Delta method was used to account for possible measurement error in both the physical activity and CVD association estimates (32, 33). The fixed-effect inverse-variance-weighted (IVW) method was implemented to evaluate the causal effect between physical activity and the outcomes. Additionally, *F* statistic was calculated to detect the strength of each instrument using the following formula: $F=\frac{R^{2}\left(N-2\right)}{\left(1-R^{2}\right)}$, where *R*^2^ stands for percentage of the variation explained by the SNPs and *N* is the sample size of the GWAS. To rule out possible pleiotropic effects, we also looked up each selected instrument SNPs in Phenoscanner (34) (http://www.phenoscanner.medschl.cam.ac.uk) to evaluate any previously reported associations (*p*-value < 5 × 10^−6^) with CVDs. Several sensitivity analyses were applied to assess robustness of the results. First, heterogeneity was evaluated by Cochran's *Q*. With a *p*-value < 0.05 indicating the presence of heterogeneity, consequently, a random-effects IVW method would be used. Second, the weighted median analysis was applied to determine invalid instrument bias. Compared to IVW, the weighted median analysis was more robust to individual genetics with strongly outlying causal estimates (35). Third, the MR-Egger regression method was used to evaluate the potential presence of directional pleiotropy based on its intercept term, where deviation from zero denotes pleiotropy (36). Fourth, MR pleiotropy residual sum and outlier (MR-PRESSO) method was also applied to identify any potential horizontal pleiotropic outliers (37). Fifth, leave-one-SNP-out analysis was used to assess whether the observed association was affected by individual SNPs.

Additionally, we also conducted multi-variable MR (MVMR) to determine the influence of potential cardiovascular risk factors on causal estimates (38). We used publicly available summary statistics for genetic association of instruments with smoking from the GWAS and Sequencing Consortium of Alcohol and Nicotine use (39), type 2 diabetes mellitus (T2D) from the Diabetes Genetics Replication and Meta-analysis (40), BMI from the Genetic Investigation of Anthropometric (41), depression from the neuroticism GWAS (42), serum lipid levels (low-density lipoprotein cholesterol and triglycerides) from the Global Lipids Genetics Consortium (43), educational attainment from the Social Science Genetic Association Consortium (44), household income from the GWAS and eQTL studies (45), and diet (vegetable and meat consumption) from BioBank Japan (46). All statistical analyses were two-sided and *p*-values < 0.05 were set as the threshold for statistical significance. All analysis was performed using R software (version 3.5.4; www.r-project.org) with the MR and MRPRESSO package.

## Results

Supplementary Table 1 showed the summarized statistics of all correlated SNPs for physical activity from the published GWAS. The *F* statistics of all SNPs were above the threshold of 10, indicating the absence of weak instrument bias. There was some evidence of heterogeneity based on Cochran's *Q* (*p*-value < 0.05) for the CVD analysis (Supplementary Table 3); consequently, for these models, the random-effects IVW method was used. In the PhenoScanner database, we identified one (rs429358, *p*-value = 4.54 × 10^−11^) of the nine SNPs for self-reported moderate-to-vigorous physical activity associated with coronary artery disease and thus we excluded it from coronary artery disease and myocardial infarction analysis (Supplementary Table 4).

### Self-Reported Physical Activity and CVDs

**Figure 1:**
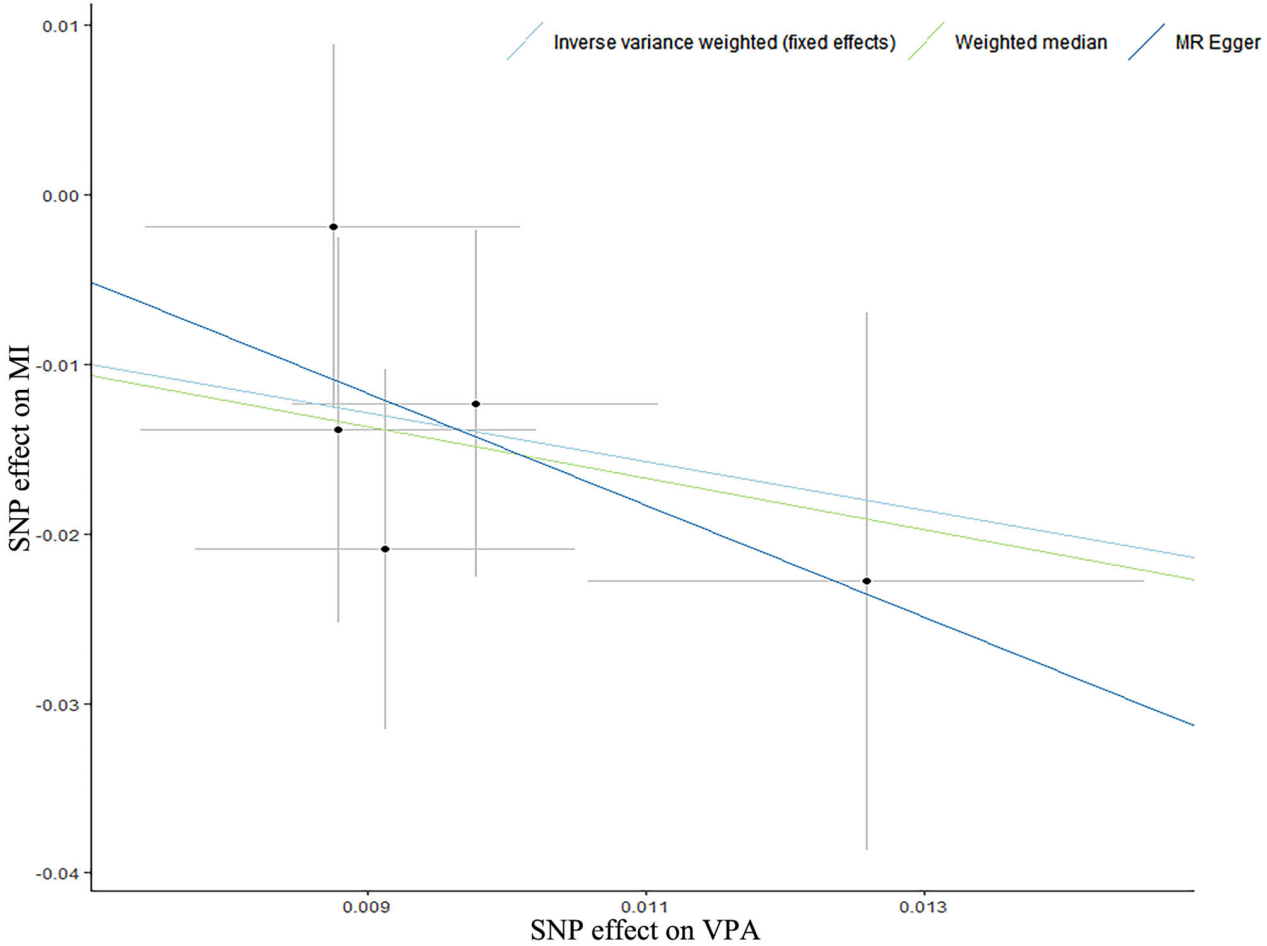
A scatter plot for the relationship of self-reported vigorous physical activity with myocardial infarction. A scatter plot of single-nucleotide polymorphism (SNP) effects regarding the associations of genetically predicted self-reported vigorous physical activity (VPA) with myocardial infarction (MI), with the slope of each line corresponding to estimated Mendelian Randomization (MR) effect per method. Circles indicate marginal genetic associations with self-reported vigorous physical activity and risk of myocardial infarction for each variant. Error bars indicate 95% CIs.

According to the IVW analysis results, we found evidence of an inverse association between VPA with myocardial infarction (IVW OR: 0.24, 95% CI: 0.08–0.68, *p*-value: 0.007), and weighted median (OR: 0.22, 95% CI: 0.06–0.78, *p*-value: 0.019) obtained a similar pattern of effect (Table 1; Figure 1). No directional pleiotropy was revealed by the MR-Egger intercept analysis (intercept, 1.02; 95% CI, 0.94–1.11; *p*-value: 0.678) (Supplementary Table 5). On the flip side, no apparent relationship was observed between VPA and heart failure, atrial fibrillation, coronary artery disease, and any ischemic stroke risk (Table 1). To explore the effect of potential cardiovascular risk factors on causal estimates, multivariable MR analysis was performed and the association of VPA with myocardial infarction was robust after adjusting for genetically predicted smoking, serum lipid levels, BMI, T2D, or depression separately (Table 2). Additionally, across all methods, we found no evidence of causal relationships of MVPA with CVDs (Table 1).

**Table 1:** Mendelian randomization estimates between self-reported physical activity and cardiovascular diseases.

**Exposure**	**Self-reported moderate-to-vigorous physical activity**					**Self-reported vigorous physical activity**				
**Outcomes**	**Method**	**OR**	**95% CI**	***p*-value**	**SNPs**	**Method**	**OR**	**95% CI**	***p*-value**	**SNPs**
Atrial fibrillation	IVW	1.13	0.79–1.6	0.508	9	IVW^§^	1.33	0.44–4.05	0.615	5
	MR-Egger	0.41	0.08–1.99	0.268	9	MR-Egger	0.00	0–13.36	0.177	5
	Weighted median	1.16	0.72–1.86	0.546	9	Weighted median	1.51	0.51–4.48	0.457	5
	MR-PRESSO	1.13	0.82–1.55	0.493	9	MR-PRESSO	1.33	0.44–4.05	0.641	5
Coronary artery disease	IVW	1.21	0.72–2.01	0.469	7^†^,^‡^	IVW	0.50	0.19–1.26	0.141	5
	MR-Egger	0.72	0.09–5.43	0.747	7^†^,^‡^	MR-Egger	0.55	0.18–1.73	0.308	5
	Weighted median	1.05	0.55–1.99	0.880	7^†^,^‡^	Weighted median	0.55	0.18–1.73	0.308	5
	MR-PRESSO	1.21	0.85–1.71	0.326	7^†^,^‡^	MR-PRESSO	0.50	0.27–0.9	0.084	5
Myocardial infarction	IVW	1.48	0.84–2.61	0.178	7^†^,^‡^	IVW	0.24	0.08–0.68	0.007	5
	MR-Egger	0.54	0–150.86	0.829	7^†^,^‡^	MR-Egger	0.04	0–271.79	0.467	5
	Weighted median	0.86	0.15–4.85	0.869	7^†^,^‡^	Weighted median	0.22	0.06–0.78	0.019	5
	MR-PRESSO	1.48	0.93–2.35	0.151	7^†^,^‡^	MR-PRESSO	0.24	0.12–0.47	0.014	5
Heart failure	IVW	0.89	0.6–1.31	0.543	9	IVW	1.57	0.71–3.45	0.263	5
	MR-Egger	0.47	0.09–2.38	0.360	9	MR-Egger	0.00	0.00–0.25	0.017	5
	Weighted median	1.10	0.64–1.87	0.738	9	Weighted median	2.24	0.74–6.77	0.151	5
	MR-PRESSO	0.89	0.62–1.27	0.532	9	MR-PRESSO	1.57	0.51–4.78	0.473	5
Ischemic stroke	IVW	1.14	0.69–1.89	0.606	8^†^	IVW	0.97	0.36–2.65	0.955	5
	MR-Egger	0.61	0.08–4.42	0.622	8^†^	MR-Egger	0.09	0.00–375.81	0.565	5
	Weighted median	1.01	0.54–1.9	0.977	8^†^	Weighted median	0.79	0.23–2.78	0.718	5
	MR-PRESSO	1.14	0.79–1.66	0.509	8^†^	MR-PRESSO	0.97	0.45–2.09	0.945	5
Large artery stroke	IVW	0.59	0.17–2.01	0.395	8^†^	IVW	4.47	0.37–54.03	0.238	5
	MR-Egger	0.07	0–34.44	0.396	8^†^	MR-Egger	32.94	0.00–10∧15	0.827	5
	Weighted median	0.47	0.09–2.51	0.374	8^†^	Weighted median	1.45	0.04–55.54	0.841	5
	MR-PRESSO	0.59	0.12–2.79	0.523	8^†^	MR-PRESSO	4.47	0.18–113.99	0.416	5
Cardioembolic stroke	IVW	1.68	0.64–4.41	0.289	8^†^	IVW	1.06	0.16–7.05	0.956	5
	MR-Egger	7.30	0.1–531.9	0.363	8^†^	MR-Egger	0.00	0.00–61073.42	0.372	5
	Weighted median	1.97	0.54–7.19	0.303	8^†^	Weighted median	0.53	0.05–5.94	0.605	5
	MR-PRESSO	1.68	0.58–4.92	0.373	8^†^	MR-PRESSO	1.06	0.1–10.86	0.966	5
Small vessel stroke	IVW	0.95	0.3–3.01	0.929	8^†^	IVW	4.16	0.41–42.13	0.228	5
	MR-Egger	0.60	0.01–60.23	0.828	8^†^	MR-Egger	0.00	0.00–18910.03	0.333	5
	Weighted median	0.85	0.19–3.87	0.832	8^†^	Weighted median	1.85	0.09–37.68	0.690	5
	MR-PRESSO	0.95	0.32–2.82	0.927	8^†^	MR-PRESSO	4.16	0.51–33.61	0.252	5

*OR, odds ratio; CI confidence intervals; IVW, inverse-variance-weighted method; MR-PRESSO, MR pleiotropy residual sum and outlier method; SNPs, single-nucleotide polymorphisms*.

§ *The estimates were evaluated from a random-effects IVW method due to the presence of heterogeneity based on Cochran's Q*.

† *rs3094622 was excluded because it was unavailable in the published GWASs and no good proxies (r^2^ > 0.8) were found*.

‡ *rs429358 was excluded since it as associated with coronary artery disease in the PhenoScanner database*.

**Table 2:** Multivariable Mendelian randomization associations of vigorous physical activity with myocardial infarction risk adjusting for cardiovascular risk factors.

**Model**	**OR**	**95% CI**	***p***
Unadjusted model	0.24	0.08–0.68	0.007
Adjusted for BMI	0.24	0.08–0.68	0.007
Adjusted for LDL-C	0.21	0.07–0.62	0.005
Adjusted for TC	0.21	0.07–0.61	0.004
Adjusted for depression	0.26	0.09–0.79	0.017
Adjusted for T2D	0.26	0.09–0.75	0.013
Adjusted for smoking	0.21	0.07–0.61	0.004
Adjusted for vegetable consumption	0.22	0.06–0.68	0.019
Adjusted for meat consumption	0.26	0.09–0.74	0.012
Adjusted for household income	0.23	0.08–0.68	0.008
Adjusted for educational attainment	0.22	0.07–0.69	0.009

*Results are scaled per genetically predicted standard deviation increase of liability to vigorous physical activity. BMI, body mass index; LDL-C, low-density lipoprotein cholesterol; TC, total cholesterol; SBP, systolic blood pressure; T2D, type 2 diabetes mellitus*.

For self-reported physical activity (including MVPA and VPA)-related SNPs, MR-PRESSO did not detect any potential outliers (Supplementary Table 6), and results of the leave-one-SNP-out analysis (Supplementary Tables 7, 8) suggested that the association between VPA and CVDs was not affected by single SNPs.

### Accelerometer-Based Physical Activity and CVDs

In contrast, there was no significant association to be found between accelerometer-based physical activity and CVDs (Table 3). These findings were confirmed using weight median, MR-Egger regression method, and leave-one-SNP-out analysis (Supplement Tables 5, 9, 10). MR-PRESSO identified several outliers (Supplementary Table 6), but similar MR estimates were observed after removal of these outliers (Supplementary Tables 11, 12).

**Table 3:** Mendelian randomization estimates between accelerometer-measured physical activity and cardiovascular diseases.

**Exposure**	**Overall acceleration average**					**Fraction of accelerations** **>** **425 milli-gravities**				
**Outcomes**	**Method**	**OR**	**95% CI**	***p*-value**	**SNPs**	**Method**	**OR**	**95% CI**	***p*-value**	**SNPs**
Atrial fibrillation	IVW^§^	1.01	0.96–1.06	0.616	8	IVW^§^	1.33	0.89–1.99	0.158	8
	MR-Egger	1.09	0.87–1.37	0.435	8	MR-Egger	20.26	0.02–10^4^	0.387	8
	Weighted median	1.00	0.96–1.04	0.947	8	Weighted median	1.09	0.78–1.53	0.595	8
	MR-PRESSO	1.01	0.96–1.06	0.631	8	MR-PRESSO	1.33	0.89–1.99	0.201	8
	MR-PRESSO (outlier-corrected)	1.00	0.97–1.03	0.871	6^#^	MR-PRESSO (outlier-corrected)	1.18	0.84–1.64	0.376	7^#^
Coronary artery disease	IVW^§^	1.02	0.97–1.08	0.396	8	IVW^§^	0.89	0.52–1.51	0.660	8
	MR-Egger	1.19	0.95–1.48	0.134	8	MR-Egger	336.74	0.08–10^6^	0.174	8
	Weighted median	1.00	0.95–1.04	0.876	8	Weighted median	0.86	0.54–1.39	0.542	8
	MR-PRESSO	1.02	0.97–1.08	0.424	8	MR-PRESSO	0.89	0.52–1.51	0.673	8
	MR-PRESSO (outlier-corrected)	1.01	0.96–1.06	0.809	7^#^	MR-PRESSO (outlier-corrected)	0.77	0.48–1.24	0.329	7^#^
Myocardial infarction	IVW^§^	1.01	0.96–1.07	0.624	8	IVW^§^	0.88	0.51–1.52	0.647	8
	MR-Egger	1.14	0.88–1.48	0.326	8	MR-Egger	720.62	0.21–2 × 10^6^	0.114	8
	Weighted median	0.99	0.94–1.04	0.792	8	Weighted median	0.67	0.41–1.1	0.113	8
	MR-presso	1.01	0.96–1.07	0.639	8	MR-PRESSO	0.88	0.51–1.52	0.661	8
	MR-presso (outlier-corrected)	0.99	0.95–1.04	0.759	7^#^	MR-PRESSO (outlier-corrected)	0.74	0.48–1.15	0.232	7^#^
Heart failure	IVW	0.98	0.95–1.01	0.132	8	IVW	1.03	0.81–1.32	0.785	8
	MR-Egger	0.99	0.85–1.17	0.945	8	MR-Egger	20.06	0.36–1,118.6	0.144	8
	Weighted median	0.98	0.95–1.02	0.413	8	Weighted median	1.00	0.72–1.38	0.985	8
	MR-PRESSO	0.98	0.95–1.01	0.293	8	MR-presso	1.03	0.82–1.31	0.783	8
	MR-PRESSO (outlier-corrected)	NA	NA	NA	NA	MR-PRESSO (outlier-corrected)	NA	NA	NA	NA
Ischemic stroke	IVW^§^	0.99	0.93–1.05	0.712	8	IVW	0.82	0.6–1.13	0.228	8
	MR-Egger	1.13	0.87–1.46	0.349	8	Simple median	0.85	0.55–1.3	0.445	8
	Weighted median	0.99	0.94–1.04	0.701	8	MR-Egger	2.19	0–1,555.14	0.815	8
	MR-PRESSO	0.99	0.93–1.05	0.723	8	MR-PRESSO	0.82	0.57–1.2	0.345	8
	MR-PRESSO (outlier-corrected)	0.97	0.92–1.03	0.378	7^#^	MR-PRESSO (outlier-corrected)	NA	NA	NA	NA
Large artery stroke	IVW	0.94	0.87–1.02	0.165	8	IVW	0.64	0.29–1.39	0.257	8
	MR-Egger	0.90	0.62–1.31	0.574	8	Simple median	0.81	0.3–2.15	0.672	8
	Weighted median	0.94	0.85–1.05	0.274	8	MR-Egger	0.02	0–6,730.82	0.538	8
	MR-PRESSO	0.94	0.9–0.99	0.054	8	MR-PRESSO	0.64	0.38–1.07	0.131	8
	MR-PRESSO (outlier-corrected)	NA	NA	NA	NA	MR-PRESSO (outlier-corrected)	NA	NA	NA	NA
Cardioembolic stroke	IVW	0.98	0.92–1.05	0.567	8	IVW	0.62	0.34–1.15	0.130	8
	MR-Egger	1.09	0.81–1.46	0.585	8	MR-Egger	1.14	0–24,321.08	0.979	8
	Weighted median	0.99	0.91–1.08	0.882	8	Weighted median	0.53	0.25–1.15	0.108	8
	MR-PRESSO	0.98	0.93–1.04	0.539	8	MR-PRESSO	0.62	0.41–0.95	0.066	8
	MR-PRESSO (outlier-corrected)	NA	NA	NA	NA	MR-PRESSO (outlier-corrected)	NA	NA	NA	NA
Small vessel stroke	IVW^§^	0.96	0.82–1.11	0.580	8	IVW	1.25	0.6–2.62	0.548	8
	MR-Egger	1.02	0.49–2.13	0.961	8	MR-Egger	10∧5	0.14–10^11^	0.094	8
	Weighted median	0.90	0.8–1.01	0.075	8	Weighted median	0.68	0.26–1.8	0.440	8
	MR-PRESSO	0.96	0.82–1.11	0.597	8	MR-PRESSO	1.25	0.49–3.2	0.651	8
	MR-PRESSO (outlier-corrected)	0.90	0.79–1.02	0.154	7^#^	MR-PRESSO (outlier-corrected)	NA	NA	NA	NA

*OR, odds ratio; CI, confidence intervals; IVW, inverse-variance-weighted method; MR-PRESSO, MR pleiotropy residual sum and outlier method; SNPs, single-nucleotide polymorphisms*.

§ *The estimates were evaluated from a random-effects IVW method due to the presence of heterogeneity based on Cochran's Q*.

# *MR-PRESSO outlier removed. Detailed information of outliers is shown in Supplementary Table 6*.

## Discussion

In this study, we applied MR to evaluate the causal relationship between physical activityand CVDs. Although physical activity might have a potential influence on the progress of CVDs, as shown in several previous studies (7–9), our study only suggests the causal relationship between self-reported vigorous physical activity and myocardial infarction.

Our findings were in line with a meta-analysis of 33 prospective cohort studies showing that physical activity was associated with myocardial infarction (7). The higher levels of physical activity, the lower risk of myocardial infarction (47). A case–control study included 18,225 individuals showing that vigorous activity is associated with a 22% lower risk of myocardial infarction (48). In this MR study, the causal relationship appeared to be motivated by self-reported vigorous physical activity, but not moderate-to-vigorous physical activity, which meant that activity levels might be more important than total activity time (49). Previous empirical studies had already suggested that several factors would affect the level of physical activity, such as education and depression (50, 51). Additionally, to obtain more robust results, we further applied MVMR analysis and demonstrated that the causal relationship would not be influenced by several CVD risk factors, including smoking, serum lipid levels, BMI, T2D, and depression. Nevertheless, there was no significant association between accelerometer-based physical activity and myocardial infarction. The discrepancy in findings between self-reported and accelerometer-based physical activity could be caused by some reasons. Firstly, compared to 377,234 participants of self-reported activity, only ~91,000 participants wore an accelerometer. Different sample sizes may influence the findings (52). Secondly, individuals who were more likely to accept wearing an accelerometer were women, aged 55–74 years, with higher socioeconomic status, and better physical health status. This may lead to selection bias and influence the results. Finally, the accelerometers only recorded the physical activity data of participants for 7 days and therefore could not effectively report the long-term activity volume.

Previous meta-analysis of observational studies reported inconsistent results on the causal relationship between physical activity (assessed using self-reported and accelerometer-measured activity) and atrial fibrillation. A large meta-analysis of 15 observational studies, including 1,464,539 participants, indicated a 6% relative reduction (hazard ratio: 0.94, 95% CI: 0.90–0.97) in atrial fibrillation risk with the guideline-recommended level of physical activity (53). In contrast, Kwok et al. conducted a meta-analysis and suggested that there is no significant decrease in AF risk with either moderate or vigorous physical activity (54). Our study may be the first to evaluate the causal association of physical activity with atrial fibrillation by using MR analysis. We found that self-reported and objective accelerometer-based physical activity was not causally associated with atrial fibrillation. These results suggested that conclusions from some previous observational studies might be false positives because of confounding and reverse causation.

Additionally, we identified no significant relationship of self-reported and objective accelerometer-based physical activity with heart failure, which supported the finding from a prospective study including 37,803 participants showing that physical activity was not independently associated with reduced risk of heart failure and indicated that the association of physical activity with incident heart failure is mediated through other risk factors, such as smoking, BMI, total cholesterol, and glucose (55). In this study, we further showed that physical activity does not affect the risk of ischemic stroke and its subtypes, which is consistent with a previous study showing that excessive activities might not be beneficial for hemorrhagic stroke (56).

Our study included several notable strengths. First, for the first time, we explored the causal relationship of self-reported and objective accelerometer-based physical activity with atrial fibrillation, heart failure, ischemic stroke, and its subtypes using MR analysis. Second, to get more robust results, we also conducted weighted median analysis, MR-Egger regression, MR-PRESSO, and leave-one-SNP-out analysis as sensitivity analysis to ensure the consistency of causal relationships. Furthermore, we also used genetically predicted objectively measured physical activity, which is more heritable than self-reported physical activity. Third, we performed multivariable MR to explore the influence of potential cardiovascular risk factors on causal estimates between VPA and myocardial infarct, and the results were consistent.

## Limitation

However, our MR study also has some limitations. First, despite the consistent results in the sensitivity analysis, we still could not completely rule out the influence of potential pleiotropy, whose physical activity might affect CVDs by other unknown causal pathways. Second, participants of physical activity in this study were all of European descent, aged 40 to 70 years, which might limit the generalizability of our findings. Further studies are needed to verify our findings in other age groups and non-European descents. Third, the instrumental variables for physical activity explained a small fraction of the phenotypic variability, which led to some results not reaching a statistical power of 80%. Consequently, the 95% confidence intervals for our MR-Egger analysis were wide in some outcomes. Thus, additional and larger replication studies were required to more robustly identify PA-associated loci. Fourth, due to the assumption of the linear relationship between exposure and outcome in MR design, a potential non-linear association of physical activity with CVDs could not be evaluated and can still not exclude the non-linear effect of physical activity.

## Conclusion

The present study supports a protective role of self-reported vigorous physical activity on the initiation of myocardial infarction and highlights the importance of activity levels of physical activity. Based on these data, the promotion of vigorous physical activity is most likely an effective strategy in the primary prevention of myocardial infarction compared with moderate-to-vigorous physical activity. Through identifying the causal relationship between physical activity and CVDs, we have laid the groundwork for future investigations on the comprehensive clinical approach in preventing the onset of cardiovascular diseases, which can be achieved through lifestyle interventions in addition to medication.

## Data Availability Statement

The summary statistics of physical activity (Study accession ID: GCST006079, GCST006097, GCST006098, and GCST006099), coronary artery disease (Study accession ID: GCST003116), myocardial infarction (Study accession ID: GCST003117), atrial fibrillation (Study accession ID: GCST006061), heart failure (Study accession ID: GCST009541), and stroke (Study accession ID: GCST005840, GCST005841, GCST005842, and GCST005843) are available at https://www.ebi.ac.uk/gwas/.

## Author Contributions

CZ: designed the study and wrote the manuscript. JZ and MC: contributed to the data analysis and data interpretation. YL: contributed to the revision of the manuscript. All authors contributed to the article and approved the submitted version.

## Funding

This work was supported by a grant from the Department of Science and Technology, Zhejiang Province (Grant no. LGF19H020011).

## Conflict of Interest

The authors declare that the research was conducted in the absence of any commercial or financial relationships that could be construed as a potential conflict of interest.

## Publisher's Note

All claims expressed in this article are solely those of the authors and do not necessarily represent those of their affiliated organizations, or those of the publisher, the editors and the reviewers. Any product that may be evaluated in this article, or claim that may be made by its manufacturer, is not guaranteed or endorsed by the publisher.
