# Supplementary material for: Physical Activity and Risks of Cardiovascular Diseases: A Mendelian Randomization Study
Source: Front Cardiovasc Med. 2021 Sep 29;8:722154. doi: 10.3389/fcvm.2021.722154 (PMC8511639; doi:10.3389/fcvm.2021.722154)
Supplement: Supplementary file 1 [file Data_Sheet_1.docx]

Supplementary Material

**Supplemental Figure 1:**Mendelian Randomization (MR) Model

**Supplemental Figure 2:** The relationship of four physical activity phenotypes

**SupplementaryTable 1.** Genome-wide significant SNPs for physical activity.

**Supplementary Tables 2.**Sample size and priori power calculations in Mendelian

**Supplemental Table 3.**Heterogeneity

**SupplementalTable 4.**Association of the SNPs used as candidate genetic instruments from the GWAS for Mendelian randomization analyses of physical activity and risk of cardiovascular disease

**Supplemental Table 5.**MR-Egger pleiotropy test

**Supplemental Table 6.**SNPs excluded from the outlier corrected MR-PRESSO analyses between physical activity and cardiovascular

**Supplementary Table 7.** Inverse variance weighted estimates for self-reported moderate-to-vigorous physical activity and cardiovascular diseases, with SNPs individually removed in leave-one-out analyses

**Supplementary Table 8.** Inverse variance weighted estimates for self-reported vigorous physical activity and cardiovascular diseases, with SNPs individually removed in leave-one-out analyses

**Supplementary Table 9.**Inverse variance weighted estimates for overall acceleration average and cardiovascular diseases, with SNPs individually removed in leave-one-out analyses

**Supplementary Table 10.** Inverse variance weighted estimates for fraction of accelerations > 425 milli-gravities and cardiovascular diseases, with SNPs individually removed in leave-one-out analyses

**Supplementary Table 11.**Mendelian Randomization estimates between overall acceleration average and cardiovascular diseases with MR-PRESSO outlier removed

**Supplementary Table 12.**Mendelian Randomization estimates between fraction of accelerations >425 milli-gravities and cardiovascular diseases with MR-PRESSO outlier removed

**Supplemental Figure 1:**Mendelian Randomization (MR) Model

**
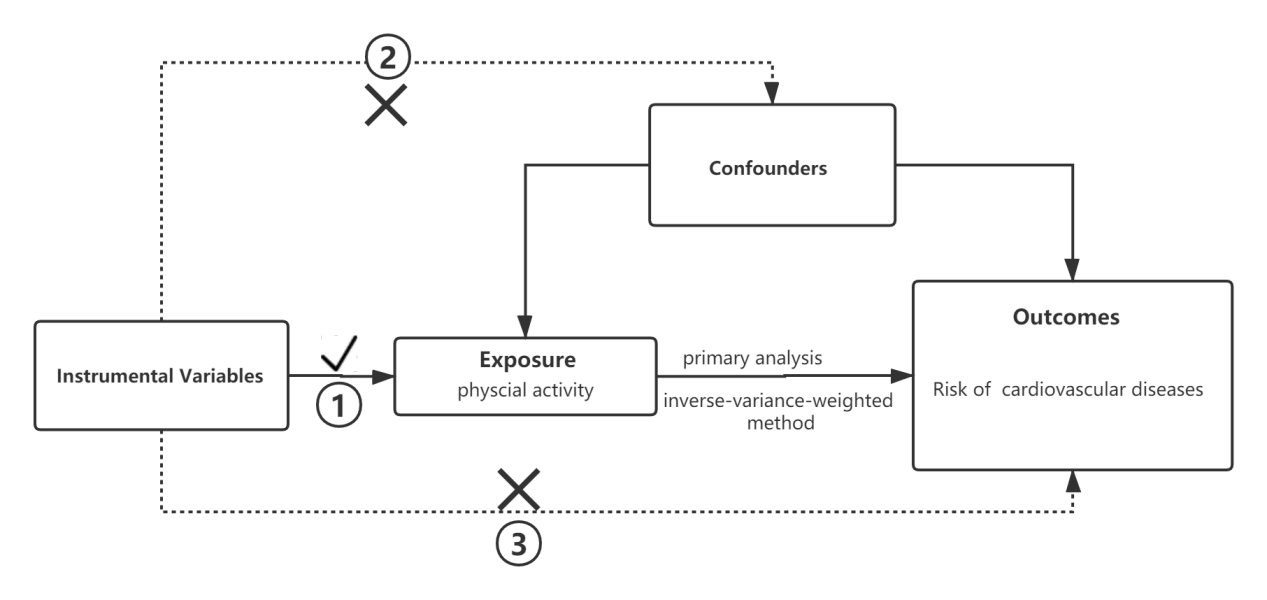
**

Figure2.A flow of the Mendelian randomization (MR) model. Three assumptions for MR are as follows: 1.the instrumental variables should be associated with the physical activity; 2.the instrumental variables must not be associated with any confounders; 3.the instrumental variables exert effects on the outcome only via the physical activity.

**Supplemental Figure 2:** The relationship of four physical activity phenotypes


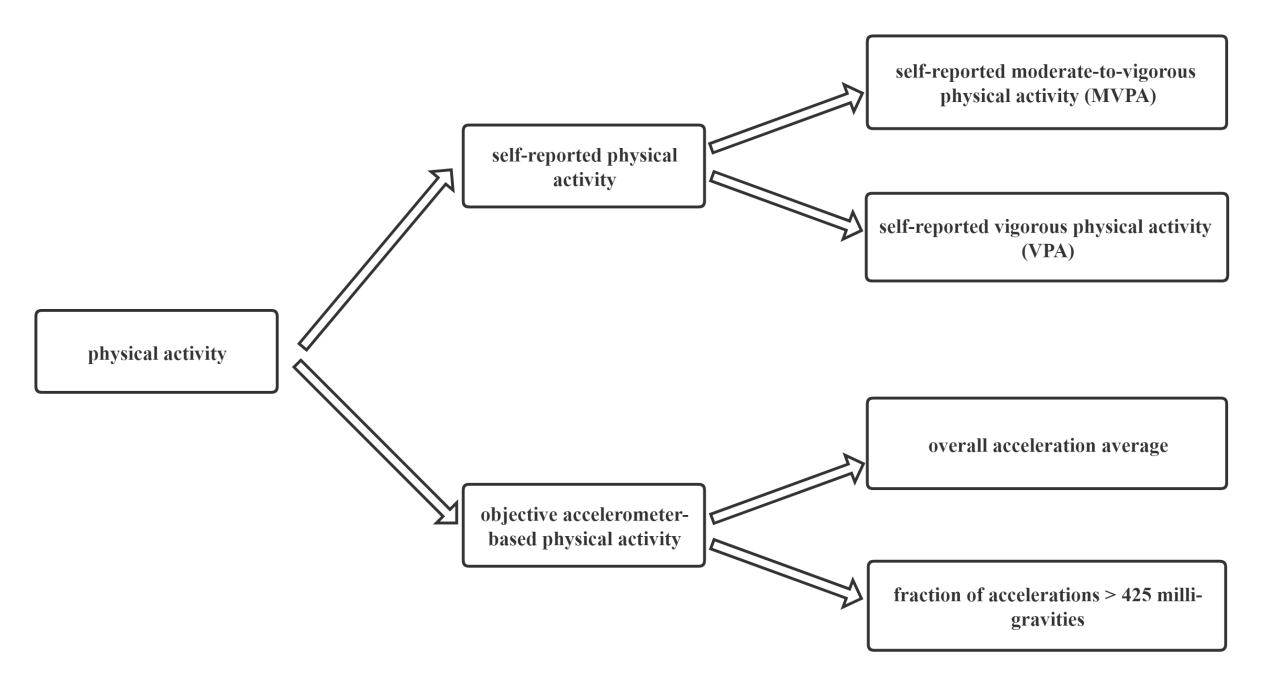


**SupplementaryTable 1.** Genome-wide significant SNPs for physical activity.

| SNP | CHR | Position | EA | OA | EAF | BETA | SE | P-value | N | R^2^ | F statistic |
| --- | --- | --- | --- | --- | --- | --- | --- | --- | --- | --- | --- |
| Overall acceleration average | | | | | | | | | | | |
| rs59499656 | 18 | 40768309 | A | T | 0.656 | -0.228 | 0.038 | 2.40E-09 | 91084 | 0.0004 | 36 |
| rs55657917 | 17 | 43844560 | T | G | 0.780 | -0.303 | 0.044 | 5.00E-12 | 91084 | 0.0005 | 48 |
| rs148193266 | 11 | 104528681 | A | C | 0.957 | -0.510 | 0.092 | 3.10E-08 | 91084 | 0.0003 | 31 |
| rs11012732 | 10 | 21830104 | A | G | 0.668 | 0.225 | 0.039 | 5.40E-09 | 91084 | 0.0004 | 34 |
| rs9293503 | 5 | 87948962 | T | C | 0.888 | 0.329 | 0.059 | 2.10E-08 | 91084 | 0.0003 | 31 |
| rs12522261 | 5 | 152054825 | G | A | 0.657 | 0.211 | 0.038 | 3.90E-08 | 91084 | 0.0003 | 30 |
| rs6775319 | 3 | 18758501 | A | T | 0.271 | 0.225 | 0.041 | 3.50E-08 | 91084 | 0.0003 | 30 |
| rs34517439 | 1 | 78450517 | C | A | 0.879 | 0.308 | 0.056 | 4.40E-08 | 91084 | 0.0003 | 30 |
| Fraction of accelerations > 425 milli-gravities | | | | | | | | | | | |
| rs1856329 | 1 | 219939623 | A | C | 0.801 | 0.027 | 0.005 | 9.00E-08 | 90667 | 0.0003 | 29 |
| rs1668835 | 18 | 22478952 | T | A | 0.688 | -0.023 | 0.004 | 3.10E-07 | 90667 | 0.0003 | 26 |
| rs80028338 | 17 | 44161470 | A | C | 0.795 | -0.028 | 0.005 | 1.50E-07 | 90667 | 0.0003 | 28 |
| rs743580 | 15 | 74328116 | A | G | 0.510 | 0.025 | 0.004 | 1.30E-09 | 90667 | 0.0004 | 37 |
| rs4754194 | 11 | 107090187 | C | T | 0.773 | -0.025 | 0.005 | 2.40E-07 | 90667 | 0.0003 | 27 |
| rs72633364 | 8 | 34186888 | G | A | 0.711 | -0.023 | 0.005 | 4.10E-07 | 90667 | 0.0003 | 26 |
| rs62443625 | 7 | 39053131 | T | C | 0.767 | -0.026 | 0.005 | 1.40E-07 | 90667 | 0.0003 | 28 |
| rs6433478 | 2 | 175241482 | T | C | 0.457 | -0.024 | 0.004 | 1.20E-08 | 90667 | 0.0004 | 32 |
| Self-reported vigorous physical activity | | | | | | | | | | | |
| rs328902 | 7 | 35020843 | C | T | 0.685 | -0.009 | 0.001 | 5.50E-10 | 261055 | 0.0001 | 38 |
| rs3781411 | 10 | 126715436 | C | T | 0.876 | 0.013 | 0.002 | 3.00E-10 | 261055 | 0.0002 | 40 |
| rs13243553 | 7 | 133506955 | G | A | 0.608 | 0.009 | 0.001 | 9.00E-11 | 261055 | 0.0002 | 42 |
| rs2764261 | 6 | 108927842 | A | G | 0.374 | 0.009 | 0.001 | 2.00E-11 | 261055 | 0.0002 | 45 |
| rs1248860 | 3 | 85015779 | G | A | 0.484 | -0.010 | 0.001 | 1.10E-13 | 261055 | 0.0002 | 55 |
| Self-reported moderate-to-vigorous physical activity | | | | | | | | | | | |
| rs3094622 | 6 | 30327952 | A | G | 0.865 | 0.020 | 0.003 | 1.40E-09 | 377234 | 0.0001 | 37 |
| rs7804463 | 7 | 133447651 | T | C | 0.530 | 0.015 | 0.002 | 1.20E-11 | 377234 | 0.0001 | 46 |
| rs7791992 | 7 | 50237784 | C | A | 0.413 | -0.014 | 0.002 | 5.70E-10 | 377234 | 0.0001 | 38 |
| rs429358 | 19 | 45411941 | T | C | 0.846 | -0.022 | 0.003 | 6.10E-13 | 377234 | 0.0001 | 52 |
| rs2988004 | 9 | 37044388 | T | G | 0.558 | -0.013 | 0.002 | 4.10E-09 | 377234 | 0.0001 | 35 |
| rs2854277 | 6 | 32628084 | C | T | 0.917 | 0.032 | 0.005 | 2.60E-10 | 377234 | 0.0001 | 40 |
| rs2035562 | 3 | 85056521 | A | G | 0.328 | -0.014 | 0.002 | 3.90E-09 | 377234 | 0.0001 | 35 |
| rs149943 | 6 | 28002388 | G | A | 0.853 | 0.019 | 0.003 | 2.20E-09 | 377234 | 0.0001 | 36 |
| rs1043595 | 7 | 128410012 | G | A | 0.717 | 0.014 | 0.002 | 4.30E-09 | 377234 | 0.0001 | 34 |

Abbreviations:SNPs, Single-nucleotide polymorphiss. CHR, chromosome. EA, effect allele. OA, other allele. EAF, effect allele frequency. SE, standard error. R², explained variation by SNPs.

**Supplementary Tables 2.**Sample size and priori power calculations in Mendelian randomization study of physical activity and risk of cardiovascular disease

| Outcomes | Exposure | Cases | Controls | Sample size | R² | OR=0.90 | OR=0.85 | OR=0.80 | OR=0.75 | OR=0.70 | OR=0.65 |
| --- | --- | --- | --- | --- | --- | --- | --- | --- | --- | --- | --- |
| Coronary artery disease | |  |  |  |  |  |  |  |  |  |  |
|  | Overall acceleration average | 60801 | 123504 | 184305 | 0.0023 | 0.17 | 0.33 | 0.55 | 0.76 | 0.9 | 0.97 |
|  | Fraction of accelerations > 425 milli-gravities | 60801 | 123504 | 184305 | 0.0023 | 0.17 | 0.33 | 0.55 | 0.76 | 0.9 | 0.97 |
|  | Self-reported moderate-to-vigorous physical activity | 60801 | 123504 | 184305 | 0.0007 | 0.09 | 0.13 | 0.21 | 0.31 | 0.44 | 0.58 |
|  | Self-reported vigorous physical activity | 60801 | 123504 | 184305 | 0.0009 | 0.1 | 0.16 | 0.26 | 0.38 | 0.53 | 0.68 |
| Myocardial infarction | |  |  |  |  |  |  |  |  |  |  |
|  | Overall acceleration average | 43676 | 128197 | 171873 | 0.0023 | 0.14 | 0.27 | 0.45 | 0.64 | 0.81 | 0.92 |
|  | Fraction of accelerations > 425 milli-gravities | 43676 | 128197 | 171873 | 0.0023 | 0.14 | 0.27 | 0.45 | 0.64 | 0.81 | 0.92 |
|  | Self-reported moderate-to-vigorous physical activity | 43676 | 128197 | 171873 | 0.0007 | 0.08 | 0.12 | 0.17 | 0.25 | 0.35 | 0.46 |
|  | Self-reported vigorous physical activity | 43676 | 128197 | 171873 | 0.0009 | 0.09 | 0.14 | 0.21 | 0.31 | 0.43 | 0.56 |
| Atrial fibrillation | |  |  |  |  |  |  |  |  |  |  |
|  | Overall acceleration average | 65446 | 522744 | 588190 | 0.0020 | 0.19 | 0.38 | 0.6 | 0.79 | 0.92 | 0.98 |
|  | Fraction of accelerations > 425 milli-gravities | 65446 | 522744 | 588190 | 0.0023 | 0.22 | 0.42 | 0.66 | 0.85 | 0.95 | 0.99 |
|  | Self-reported moderate-to-vigorous physical activity | 65446 | 522744 | 588190 | 0.0009 | 0.11 | 0.2 | 0.32 | 0.46 | 0.62 | 0.76 |
|  | Self-reported vigorous physical activity | 65446 | 522744 | 588190 | 0.0009 | 0.11 | 0.2 | 0.32 | 0.46 | 0.62 | 0.76 |
| Heart failure | |  |  |  |  |  |  |  |  |  |  |
|  | Overall acceleration average | 47309 | 930014 | 977323 | 0.0028 | 0.2 | 0.4 | 0.62 | 0.81 | 0.93 | 0.98 |
|  | Fraction of accelerations > 425 milli-gravities | 47309 | 930014 | 977323 | 0.0026 | 0.19 | 0.37 | 0.59 | 0.78 | 0.91 | 0.97 |
|  | Self-reported moderate-to-vigorous physical activity | 47309 | 930014 | 977323 | 0.0009 | 0.1 | 0.16 | 0.25 | 0.36 | 0.49 | 0.62 |
|  | Self-reported vigorous physical activity | 47309 | 930014 | 977323 | 0.0009 | 0.1 | 0.16 | 0.25 | 0.36 | 0.49 | 0.62 |
| Ischemic stroke | |  |  |  |  |  |  |  |  |  |  |
|  | Overall acceleration average | 34217 | 406111 | 440328 | 0.0025 | 0.15 | 0.27 | 0.44 | 0.62 | 0.78 | 0.9 |
|  | Fraction of accelerations > 425 milli-gravities | 34217 | 406111 | 440328 | 0.0026 | 0.15 | 0.28 | 0.45 | 0.64 | 0.8 | 0.91 |
|  | Self-reported moderate-to-vigorous physical activity | 34217 | 406111 | 440328 | 0.0008 | 0.08 | 0.12 | 0.18 | 0.25 | 0.34 | 0.44 |
|  | Self-reported vigorous physical activity | 34217 | 406111 | 440328 | 0.0009 | 0.08 | 0.13 | 0.19 | 0.28 | 0.38 | 0.49 |
| Large artery stroke | |  |  |  |  |  |  |  |  |  |  |
|  | Overall acceleration average | 4373 | 146392 | 150765 | 0.0028 | 0.06 | 0.08 | 0.11 | 0.14 | 0.18 | 0.23 |
|  | Fraction of accelerations > 425 milli-gravities | 4373 | 146392 | 150765 | 0.0026 | 0.06 | 0.08 | 0.1 | 0.13 | 0.17 | 0.22 |
|  | Self-reported moderate-to-vigorous physical activity | 4373 | 146392 | 150765 | 0.0008 | 0.05 | 0.06 | 0.07 | 0.08 | 0.09 | 0.1 |
|  | Self-reported vigorous physical activity | 4373 | 146392 | 150765 | 0.0009 | 0.05 | 0.06 | 0.07 | 0.08 | 0.09 | 0.11 |
| Cardioembolic stroke | |  |  |  |  |  |  |  |  |  |  |
|  | Overall acceleration average | 7193 | 204570 | 211763 | 0.0028 | 0.07 | 0.1 | 0.14 | 0.2 | 0.27 | 0.35 |
|  | Fraction of accelerations > 425 milli-gravities | 7193 | 204570 | 211763 | 0.0026 | 0.07 | 0.1 | 0.14 | 0.19 | 0.25 | 0.33 |
|  | Self-reported moderate-to-vigorous physical activity | 7193 | 204570 | 211763 | 0.0008 | 0.06 | 0.06 | 0.08 | 0.09 | 0.11 | 0.13 |
|  | Self-reported vigorous physical activity | 7193 | 204570 | 211763 | 0.0009 | 0.06 | 0.07 | 0.08 | 0.1 | 0.12 | 0.14 |
| Small vessel stroke | |  |  |  |  |  |  |  |  |  |  |
|  | Overall acceleration average | 5386 | 192662 | 198048 | 0.0023 | 0.06 | 0.08 | 0.11 | 0.14 | 0.18 | 0.23 |
|  | Fraction of accelerations > 425 milli-gravities | 5386 | 192662 | 198048 | 0.0026 | 0.07 | 0.09 | 0.11 | 0.15 | 0.2 | 0.26 |
|  | Self-reported moderate-to-vigorous physical activity | 5386 | 192662 | 198048 | 0.0008 | 0.05 | 0.06 | 0.07 | 0.08 | 0.09 | 0.11 |
|  | Self-reported vigorous physical activity | 5386 | 192662 | 198048 | 0.0009 | 0.06 | 0.06 | 0.07 | 0.08 | 0.1 | 0.12 |

R², explained variation by SNPs. OR, odds ratio.

**Supplemental Table 3.**Heterogeneity

| Physical activity | Outcome | Cochran's Q | Degrees of Freedom | P-value |
| --- | --- | --- | --- | --- |
| Overall acceleration average | | | | |
|  | Atrial fibrillation | 5.053 | 5 | 0.4095 |
|  | Coronary artery disease | 11.216 | 6 | 0.0819 |
|  | Myocardial infarction | 8.365 | 6 | 0.2126 |
|  | Heart failure | 12.250 | 7 | 0.0926 |
|  | Ischemic stroke | 16.839 | 6 | 0.0099 |
|  | Large artery stroke | 2.532 | 7 | 0.9247 |
|  | Cardioembolic stroke | 5.516 | 7 | 0.5972 |
|  | Small vessel stroke | 13.730 | 6 | 0.0328 |
| Fraction of accelerations > 425 milli-gravities | | | | |
|  | Atrial fibrillation | 11.501 | 6 | 0.0741 |
|  | Coronary artery disease | 13.682 | 6 | 0.0334 |
|  | Myocardial infarction | 2.566 | 6 | 0.8610 |
|  | Heart failure | 6.372 | 7 | 0.4970 |
|  | Ischemic stroke | 9.896 | 7 | 0.1945 |
|  | Large artery stroke | 3.067 | 7 | 0.8788 |
|  | Cardioembolic stroke | 3.391 | 7 | 0.8467 |
|  | Small vessel stroke | 11.345 | 7 | 0.1243 |
| Self-reported vigorous physical activity | | | | |
|  | Atrial fibrillation | 6.806 | 8 | 0.5577 |
|  | Coronary artery disease | 3.211 | 6 | 0.8648 |
|  | Myocardial infarction | 4.681 | 6 | 0.6989 |
|  | Heart failure | 6.917 | 8 | 0.5456 |
|  | Ischemic stroke | 4.389 | 7 | 0.8205 |
|  | Large artery stroke | 12.812 | 7 | 0.1185 |
|  | Cardioembolic stroke | 9.912 | 7 | 0.2712 |
|  | Small vessel stroke | 7.116 | 7 | 0.5242 |
| Self-reported moderate-to-vigorous physical activity | | | | |
|  | Atrial fibrillation | 9.709 | 4 | 0.0456 |
|  | Coronary artery disease | 1.653 | 4 | 0.7992 |
|  | Myocardial infarction | 1.662 | 4 | 0.7977 |
|  | Heart failure | 8.015 | 4 | 0.0910 |
|  | Ischemic stroke | 2.326 | 4 | 0.6761 |
|  | Large artery stroke | 6.756 | 4 | 0.1493 |
|  | Cardioembolic stroke | 6.026 | 4 | 0.1972 |
|  | Small vessel stroke | 3.257 | 4 | 0.5158 |

**Supplemental Table 4.**Association of the SNPs used as candidate genetic instruments from the GWAS for Mendelian randomization analyses of physical activity and risk of cardiovascular disease

| SNP | CHR | Position | Diseases & traits | Excluded from MR analysis |
| --- | --- | --- | --- | --- |
| Overall acceleration average | | | | |
| rs59499656 | 18 | 40768309 | body fat percentage/mass(UKBB), trunk fat percentage/mass (UKBB), arm fat percentage/mass(UKBB),leg fat percentage/mass(UKBB),weight(UKBB),body mass index(UKBB) | NO |
| rs55657917 | 17 | 43844560 | height(UKBB),Systolic blood pressure(UKBB) | NO |
| rs148193266 | 11 | 104528681 | N/A | NO |
| rs11012732 | 10 | 21830104 | leg fat mass/percentage(UKBB), waist circumference (UKBB), hip circumference(UKBB), body mass index (UKBB), weight (UKBB), arm fat mass(UKBB), trunk fat(UKBB), diastolic blood pressure(UKBB), past tobacco smoking(UKBB) | NO |
| rs9293503 | 5 | 87948962 | trunk fat-free/predicted mass (UKBB), arm fat-free/predicted mass (UKBB), leg fat percentage/mass (UKBB),weight(UKBB),self-reported hypertension(UKBB) | NO |
| rs12522261 | 5 | 152054825 | N/A | NO |
| rs6775319 | 3 | 18758501 | body fat percentage(UKBB), trunk fat percentage/mass (UKBB), arm fat percentage/mass (UKBB), leg fat percentage/mass (UKBB),weight(UKBB),body mass index(UKBB), whole body fat mass(UKBB) | NO |
| rs34517439 | 1 | 78450517 | diastolic blood pressure (PMID: 30224653),Hip circumference (UKBB), waist circumference (UKBB),Height (UKBB), weight (UKBB), body mass index (UKBB), leg fat-free mass(UKBB), leg predicted mass(UKBB),arm fat mass(UKBB), leg fat-free mass(UKBB) | NO |
| Fraction of accelerations > 425 milli-gravities | | | | |
| rs1856329 | 1 | 219939623 | N/A | NO |
| rs1668835 | 18 | 22478952 | N/A | NO |
| rs80028338 | 17 | 44161470 | alcohol intake frequency(UKBB), height(UKBB), systolic blood pressure(UKBB), trunk fat mass(UKBB) | NO |
| rs743580 | 15 | 74328116 | height(UKBB), leg fat(UKBB), body mass index(UKBB), arm fat(UKBB), body fat(UKBB), waist circumference(UKBB) | NO |
| rs4754194 | 11 | 107090187 | systolic blood pressure(UKBB) | NO |
| rs72633364 | 8 | 34186888 | N/A | NO |
| rs62443625 | 7 | 39053131 | trunk fat percentage(UKBB), arm fat percentage (UKBB), body fat percentage(UKBB),whole body fat mass(UKBB) | NO |
| rs6433478 | 2 | 175241482 | hand grip strength left(UKBB) | NO |
| Self-reported vigorous physical activity | | | | |
| rs328902 | 7 | 35020843 | impedance of arm left(UKBB), impedance of whole body(UKBB) | NO |
| rs3781411 | 10 | 126715436 | N/A | NO |
| rs13243553 | 7 | 133506955 | N/A | NO |
| rs2764261 | 6 | 108927842 | trunk fat-free/predicted mass(UKBB), arm fat-free/predicted mass(UKBB), leg fat-free/predicted mass(UKBB), weight(UKBB), height(UKBB), body mass index (UKBB) | NO |
| rs1248860 | 3 | 85015779 | trunk fat-free/predicted mass(UKBB), arm fat-free/predicted mass(UKBB), leg fat-free/predicted mass(UKBB), weight(UKBB), height(UKBB), body mass index (PMID:29273807), smoking status: previous(UKBB) | NO |
| Self-reported moderate-to-vigorous physical activity | | | | |
| rs3094622 | 6 | 30327952 | treatment with insulin product(UKBB), height(UKBB), trunk fat-free/predicted mass(UKBB), arm fat-free/predicted mass(UKBB), leg fat-free/predicted mass(UKBB), weight(UKBB), triglycerides(PMID:24097068) | NO |
| rs7804463 | 7 | 133447651 | N/A | NO |
| rs7791992 | 7 | 50237784 | N/A | NO |
| rs429358 | 19 | 45411941 | self-reported high cholesterol(UKBB), coronary artery disease(PMID:29212778), waistcircumference(UKBB), body mass index (UKBB), arm fat-free/predicted mass(UKBB), leg fat-free/predicted mass(UKBB) | YES(CAD,MI) |
| rs2988004 | 9 | 37044388 | N/A | NO |
| rs2854277 | 6 | 32628084 | N/A | NO |
| rs2035562 | 3 | 85056521 | trunk fat-free/predicted mass(UKBB), arm fat-free/predicted mass(UKBB), leg fat-free/predicted mass(UKBB), weight(UKBB), body mass index (UKBB), past tobacco smoking(UKBB) | NO |
| rs149943 | 6 | 28002388 | diastolic blood pressure(UKBB), weight(UKBB), hip circumference(UKBB),Trunk fat percentage/mass(UKBB), leg fat-free/predicted mass(UKBB) | NO |
| rs1043595 | 7 | 128410012 | height(PMID:25282103) | NO |

SNP,single-nucleotide polymorphism.CHR, chromosome.MR,mendelian randomization. UKBB, UK biobank.

**Supplemental Table 5.**MR-Egger pleiotropy test

| Outcomes | method | OR | 95%CI | P-valve | SNPs |
| --- | --- | --- | --- | --- | --- |
| Overall acceleration average | | | | | |
| AF | MR-Egger | 1.09 | 0.87-1.37 | 0.435 | 8 |
|  | (intercept) | 0.98 | 0.92-1.04 | 0.490 | 8 |
|  | MR-Egger* | 1.09 | 0.96-1.22 | 0.182 | 6 |
|  | (intercept)* | 0.98 | 0.95-1.01 | 0.183 | 6 |
| CAD | MR-Egger | 1.19 | 0.95-1.48 | 0.134 | 8 |
|  | (intercept) | 0.96 | 0.91-1.02 | 0.183 | 8 |
|  | MR-Egger* | 1.14 | 0.94-1.38 | 0.196 | 7 |
|  | (intercept)* | 0.97 | 0.92-1.02 | 0.205 | 7 |
| MI | MR-Egger | 1.14 | 0.88-1.48 | 0.326 | 8 |
|  | (intercept) | 0.97 | 0.91-1.04 | 0.370 | 8 |
|  | MR-Egger* | 1.08 | 0.88-1.32 | 0.477 | 7 |
|  | (intercept)* | 0.98 | 0.93-1.03 | 0.420 | 7 |
| HF | MR-Egger | 0.99 | 0.85-1.17 | 0.945 | 8 |
|  | (intercept) | 1.00 | 0.95-1.04 | 0.857 |  |
| IS | MR-Egger | 1.13 | 0.87-1.46 | 0.349 | 8 |
|  | (intercept) | 0.96 | 0.9-1.03 | 0.294 | 8 |
|  | MR-Egger* | 0.61 | 0-422087.07 | 0.943 | 7 |
|  | (intercept)* | 1.01 | 0.73-1.4 | 0.957 | 7 |
| LAS | MR-Egger | 0.90 | 0.62-1.31 | 0.574 | 8 |
|  | (intercept) | 1.01 | 0.92-1.12 | 0.794 | 8 |
| CS | MR-Egger | 1.09 | 0.81-1.46 | 0.585 | 8 |
|  | (intercept) | 0.97 | 0.9-1.05 | 0.490 | 8 |
| SVS | MR-Egger | 1.02 | 0.49-2.13 | 0.961 | 8 |
|  | (intercept) | 0.98 | 0.81-1.19 | 0.868 | 8 |
|  | MR-Egger* | 0.86 | 0.48-1.56 | 0.622 | 7 |
|  | (intercept)* | 1.01 | 0.87-1.18 | 0.881 | 7 |
| Fraction of accelerations > 425 milli-gravities | | | | | |
| AF | MR-Egger | 20.26 | 0.02-18564.63 | 0.387 | 8 |
|  | (intercept) | 0.93 | 0.79-1.11 | 0.434 | 8 |
|  | MR-Egger* | 0.56 | 0.00-494.04 | 0.866 | 7 |
|  | (intercept)* | 1.02 | 0.86-1.2 | 0.829 | 7 |
| CAD | MR-Egger | 336.74 | 0.08-1468272.21 | 0.174 | 8 |
|  | (intercept) | 0.86 | 0.7-1.06 | 0.164 | 8 |
|  | MR-Egger* | 16.94 | 0-182893.82 | 0.550 | 7 |
|  | (intercept)* | 0.93 | 0.74-1.17 | 0.514 | 7 |
| MI | MR-Egger | 720.62 | 0.21-2495576.54 | 0.114 | 8 |
|  | (intercept) | 0.85 | 0.69-1.04 | 0.106 | 8 |
|  | MR-Egger* | 11.79 | 0-887584.36 | 0.667 | 7 |
|  | (intercept)* | 0.93 | 0.71-1.23 | 0.619 | 7 |
| HF | MR-Egger | 20.06 | 0.36-1118.57 | 0.144 | 8 |
|  | (intercept) | 0.93 | 0.84-1.03 | 0.148 | 8 |
| IS | MR-Egger | 2.19 | 0-1555.14 | 0.815 | 8 |
|  | (intercept) | 0.98 | 0.83-1.15 | 0.770 | 8 |
| LAS | MR-Egger | 0.02 | 0-6730.82 | 0.538 | 8 |
|  | (intercept) | 1.09 | 0.79-1.5 | 0.584 | 8 |
| CS | MR-Egger | 1.14 | 0-24321.08 | 0.979 | 8 |
|  | (intercept) | 0.99 | 0.77-1.26 | 0.905 | 8 |
| SVS | MR-Egger | 121870.57 | 0.14-10^11 | 0.094 | 8 |
|  | (intercept) | 0.75 | 0.53-1.06 | 0.099 | 8 |
| Self-reported moderate-to-vigorous physical activity | | | | | |
| AF | MR-Egger | 0.41 | 0.08-1.99 | 0.268 | 9 |
|  | (intercept) | 1.02 | 0.99-1.04 | 0.175 | 9 |
| CAD | MR-Egger | 0.72 | 0.09-5.43 | 0.747 | 7 |
|  | (intercept) | 1.01 | 0.98-1.04 | 0.602 | 7 |
| MI | MR-Egger | 0.54 | 0-150.86 | 0.829 | 7 |
|  | (intercept) | 1.01 | 0.92-1.1 | 0.840 | 7 |
| HF | MR-Egger | 0.47 | 0.09-2.38 | 0.360 | 9 |
|  | (intercept) | 1.01 | 0.98-1.04 | 0.423 | 9 |
| IS | MR-Egger | 0.61 | 0.08-4.42 | 0.622 | 8 |
|  | (intercept) | 1.01 | 0.98-1.04 | 0.519 | 8 |
| LAS | MR-Egger | 0.07 | 0-34.44 | 0.396 | 8 |
|  | (intercept) | 1.04 | 0.94-1.15 | 0.481 | 8 |
| CS | MR-Egger | 7.30 | 0.1-531.9 | 0.363 | 8 |
|  | (intercept) | 0.98 | 0.91-1.05 | 0.487 | 8 |
| SVS | MR-Egger | 0.60 | 0.01-60.23 | 0.828 | 8 |
|  | (intercept) | 1.01 | 0.93-1.09 | 0.840 | 8 |
| Self-reported vigorous physical activity | | | | | |
| AF | MR-Egger | 0.00 | 0-13.36 | 0.177 | 5 |
|  | (intercept) | 1.06 | 0.98-1.15 | 0.154 | 5 |
| CAD | MR-Egger | 0.55 | 0.18-1.73 | 0.308 | 5 |
|  | (intercept) | 0.99 | 0.92-1.07 | 0.829 | 5 |
| MI | MR-Egger | 0.04 | 0-271.79 | 0.467 | 5 |
|  | (intercept) | 1.02 | 0.94-1.11 | 0.678 | 5 |
| HF | MR-Egger | 0.00 | 0.00-0.25 | 0.017 | 5 |
|  | (intercept) | 1.08 | 1.02-1.15 | 0.011 | 5 |
| IS | MR-Egger | 0.09 | 0.00-375.81 | 0.565 | 5 |
|  | (intercept) | 1.02 | 0.94-1.11 | 0.567 | 5 |
| LAS | MR-Egger | 32.94 | 0.00-10^15 | 0.827 | 5 |
|  | (intercept) | 0.98 | 0.73-1.32 | 0.900 | 5 |
| CS | MR-Egger | 0.00 | 0.00-61073.42 | 0.372 | 5 |
|  | (intercept) | 1.09 | 0.9-1.33 | 0.366 | 5 |
| SVS | MR-Egger | 0.00 | 0.00-18910.03 | 0.333 | 5 |
|  | (intercept) | 1.11 | 0.92-1.34 | 0.263 | 5 |

OR, odds ratio.CI confidence intervals.SNPs, single-nucleotide polymorphisms. AF, atrial fibrillation. CAD, coronary artery disease. MI, myocardial infarction. HF,heart failure. IS, ischemic stroke. LAS, large artery stroke. CS, cardioembolic stroke. SVS, Small vessel stroke.

*model with MR-PRESSO outliers removed.Detailed information of outliers were shown in Supplemental Table 6.

**Supplemental Table 6.** SNPs excluded from the outlier corrected MR-PRESSO analyses between physical activity and cardiovascular

| Physcial activity | Outcome | OutlierSNP | RSSobs | P-value |
| --- | --- | --- | --- | --- |
| Overall acceleration average | | | | |
|  | AF | rs34517439 | 0.001359286 | 0.016 |
|  |  | rs113871181 | 0.001753247 | <0.008 |
|  | CAD | rs113871181 | 0.001542932 | 0.016 |
|  | MI | rs113871181 | 0.002408828 | <0.008 |
|  | HF | NA | NA | NA |
|  | IS | rs9293503 | 0.00194977 | 0.048 |
|  | CS | NA | NA | NA |
|  | LAS | NA | NA | NA |
|  | SVS | rs113871181 | 1.33E-02 | 0.008 |
| Fraction of accelerations > 425 milli-gravities | | | | |
|  | AF | rs80028338 | 1.11E-03 | 0.008 |
|  | CAD | rs80028338 | 1.78E-03 | 0.016 |
|  | MI | rs80028338 | 2.32E-03 | 0.008 |
|  | HF | NA | NA | NA |
|  | IS | NA | NA | NA |
|  | CS | NA | NA | NA |
|  | LAS | NA | NA | NA |
|  | SVS | NA | NA | NA |
| Self-reported vigorous physical activity | | | | |
|  | NA | NA | NA | NA |
| Self-reported moderate-to-vigorous physical activity | | | | |
|  | NA | NA | NA | NA |

Excluded variants are shown for the analyses between four physical activity phenotypes and cardiovascular diseases. AF, atrial fibrillation. CAD, coronary artery disease. MI, myocardial infarction. HF,heart failure. IS, ischemic stroke. LAS, large artery stroke. CS, cardioembolic stroke. SVS, Small vessel stroke.OutlierSNP, Single nucleotide polymorphism removed from the analysis by MR-PRESSO. RSSobs, observed residual sum of squares.

**Supplementary Table 7.** Inverse variance weighted estimates for self-reported moderate-to-vigorous physical activity and cardiovascular diseases, with SNPs individually removed in leave-one-out analyses

| Outcome | Excluded SNP | OR | 95%CI | P-value | P-value for heterogeneity |
| --- | --- | --- | --- | --- | --- |
| CAD |  |  |  |  |  |
|  | rs3094622^†^ | NA | NA | NA | NA |
|  | rs7804463 | 2.24 | 0.70-7.15 | 0.172 | <0.001 |
|  | rs7791992 | 2.32 | 0.76-7.07 | 0.141 | <0.001 |
|  | rs2988004 | 1.90 | 0.60-6.01 | 0.277 | <0.001 |
|  | rs2854277 | 2.31 | 0.72-7.37 | 0.157 | <0.001 |
|  | rs2035562 | 2.13 | 0.67-6.74 | 0.198 | <0.001 |
|  | rs149943 | 2.03 | 0.65-6.40 | 0.224 | <0.001 |
| MI |  |  |  |  |  |
|  | rs3094622^†^ | NA | NA | NA | NA |
|  | rs7804463 | 2.68 | 0.79-9.06 | 0.113 | <0.001 |
|  | rs7791992 | 2.92 | 0.93-9.20 | 0.067 | <0.001 |
|  | rs2988004 | 2.25 | 0.68-7.42 | 0.183 | <0.001 |
|  | rs2854277 | 2.58 | 0.74-8.99 | 0.137 | <0.001 |
|  | rs2035562 | 2.88 | 0.92-9.07 | 0.070 | <0.001 |
|  | rs149943 | 2.48 | 0.75-8.19 | 0.138 | <0.001 |
| AF |  |  |  |  |  |
|  | rs3094622 | 1.19 | 0.82-1.71 | 0.363 | 0.543 |
|  | rs7804463 | 1.12 | 0.77-1.63 | 0.556 | 0.450 |
|  | rs7791992 | 1.16 | 0.80-1.69 | 0.425 | 0.478 |
|  | rs429358 | 1.19 | 0.82-1.74 | 0.360 | 0.520 |
|  | rs2988004 | 0.99 | 0.68-1.43 | 0.956 | 0.940 |
|  | rs2854277 | 1.18 | 0.82-1.71 | 0.372 | 0.527 |
|  | rs2035562 | 1.12 | 0.77-1.62 | 0.557 | 0.451 |
|  | rs149943 | 1.07 | 0.74-1.55 | 0.709 | 0.523 |
|  | rs1043595 | 1.12 | 0.78-1.62 | 0.539 | 0.450 |
| HF |  |  |  |  |  |
|  | rs3094622 | 0.89 | 0.60-1.33 | 0.568 | 0.438 |
|  | rs7804463 | 0.85 | 0.56-1.29 | 0.437 | 0.472 |
|  | rs7791992 | 0.96 | 0.63-1.45 | 0.838 | 0.566 |
|  | rs429358 | 1.04 | 0.69-1.58 | 0.848 | 0.872 |
|  | rs2988004 | 0.81 | 0.54-1.22 | 0.311 | 0.654 |
|  | rs2854277 | 0.85 | 0.56-1.29 | 0.449 | 0.466 |
|  | rs2035562 | 0.85 | 0.56-1.27 | 0.420 | 0.493 |
|  | rs149943 | 0.89 | 0.60-1.33 | 0.579 | 0.439 |
|  | rs1043595 | 0.87 | 0.58-1.31 | 0.516 | 0.443 |
| IS |  |  |  |  |  |
|  | rs3094622^†^ | NA | NA | NA | NA |
|  | rs7804463 | 1.18 | 0.68-2.04 | 0.552 | 0.736 |
|  | rs7791992 | 1.26 | 0.73-2.16 | 0.402 | 0.859 |
|  | rs429358 | 1.18 | 0.68-2.03 | 0.564 | 0.733 |
|  | rs2988004 | 1.04 | 0.61-1.78 | 0.873 | 0.850 |
|  | rs2854277 | 1.16 | 0.67-2.01 | 0.602 | 0.725 |
|  | rs2035562 | 1.05 | 0.61-1.79 | 0.860 | 0.835 |
|  | rs149943 | 1.22 | 0.72-2.07 | 0.455 | 0.825 |
|  | rs1043595 | 1.07 | 0.63-1.83 | 0.805 | 0.796 |
| CS |  |  |  |  |  |
|  | rs3094622^†^ | NA | NA | NA | NA |
|  | rs7804463 | 1.71 | 0.60-4.87 | 0.317 | 0.182 |
|  | rs7791992 | 2.05 | 0.73-5.74 | 0.172 | 0.268 |
|  | rs429358 | 2.30 | 0.81-6.49 | 0.117 | 0.402 |
|  | rs2988004 | 1.56 | 0.56-4.32 | 0.394 | 0.195 |
|  | rs2854277 | 1.27 | 0.44-3.67 | 0.655 | 0.307 |
|  | rs2035562 | 2.07 | 0.74-5.75 | 0.164 | 0.287 |
|  | rs149943 | 1.33 | 0.49-3.64 | 0.577 | 0.416 |
|  | rs1043595 | 1.48 | 0.53-4.08 | 0.451 | 0.225 |
| SVS |  |  |  |  |  |
|  | rs3094622^†^ | NA | NA | NA | NA |
|  | rs7804463 | 0.67 | 0.19-2.36 | 0.537 | 0.656 |
|  | rs7791992 | 1.40 | 0.41-4.81 | 0.595 | 0.810 |
|  | rs429358 | 0.95 | 0.27-3.31 | 0.931 | 0.402 |
|  | rs2988004 | 1.05 | 0.31-3.58 | 0.934 | 0.433 |
|  | rs2854277 | 1.01 | 0.29-3.55 | 0.992 | 0.408 |
|  | rs2035562 | 0.77 | 0.23-2.63 | 0.676 | 0.528 |
|  | rs149943 | 1.08 | 0.32-3.62 | 0.906 | 0.456 |
|  | rs1043595 | 0.83 | 0.24-2.82 | 0.766 | 0.454 |
| LAS |  |  |  |  |  |
|  | rs3094622^†^ | NA | NA | NA | NA |
|  | rs7804463 | 0.81 | 0.21-3.09 | 0.758 | 0.126 |
|  | rs7791992 | 0.44 | 0.12-1.64 | 0.220 | 0.126 |
|  | rs429358 | 0.47 | 0.12-1.78 | 0.265 | 0.094 |
|  | rs2988004 | 0.61 | 0.16-2.25 | 0.456 | 0.071 |
|  | rs2854277 | 0.70 | 0.18-2.71 | 0.602 | 0.081 |
|  | rs2035562 | 0.37 | 0.10-1.39 | 0.142 | 0.304 |
|  | rs149943 | 0.90 | 0.24-3.28 | 0.868 | 0.336 |
|  | rs1043595 | 0.59 | 0.16-2.16 | 0.423 | 0.070 |

^†^SNPs were excluded since they were unavailable in the GWAS and no good proxies (r^2^>0.8) were found. OR, odds ratio.CI confidence intervals.SNPs, single-nucleotide polymorphisms. AF, atrial fibrillation. CAD, coronary artery disease. MI, myocardial infarction. HF,heart failure. IS, ischemic stroke. LAS, large artery stroke. CS, cardioembolic stroke. SVS, Small vessel stroke.

**Supplementary Table 8.** Inverse variance weighted estimates for self-reported vigorous physical activity and cardiovascular diseases, with SNPs individually removed in leave-one-out analyses

| Outcome | Excluded SNP | OR | 95%CI | P-value | P-value for heterogeneity |
| --- | --- | --- | --- | --- | --- |
| CAD |  |  |  |  |  |
|  | rs328902 | 0.64 | 0.23-1.77 | 0.386 | 0.961 |
|  | rs3781411 | 0.51 | 0.18-1.42 | 0.197 | 0.650 |
|  | rs13243553 | 0.50 | 0.18-1.42 | 0.192 | 0.648 |
|  | rs2764261 | 0.44 | 0.15-1.24 | 0.120 | 0.714 |
|  | rs1248860 | 0.42 | 0.14-1.23 | 0.114 | 0.736 |
| MI |  |  |  |  |  |
|  | rs328902 | 0.25 | 0.08-0.77 | 0.016 | 0.649 |
|  | rs3781411 | 0.26 | 0.08-0.82 | 0.021 | 0.671 |
|  | rs13243553 | 0.18 | 0.06-0.57 | 0.004 | 0.930 |
|  | rs2764261 | 0.30 | 0.09-0.97 | 0.044 | 0.808 |
|  | rs1248860 | 0.23 | 0.07-0.76 | 0.016 | 0.653 |
| AF |  |  |  |  |  |
|  | rs328902 | 1.12 | 0.30-4.20 | 0.870 | 0.036 |
|  | rs3781411 | 1.96 | 0.89-4.33 | 0.096 | 0.188 |
|  | rs13243553 | 1.65 | 0.44-6.15 | 0.455 | 0.042 |
|  | rs2764261 | 0.90 | 0.41-2.01 | 0.807 | 0.152 |
|  | rs1248860 | 1.27 | 0.29-5.58 | 0.754 | 0.022 |
| HF |  |  |  |  |  |
|  | rs328902 | 1.24 | 0.52-2.94 | 0.633 | 0.097 |
|  | rs3781411 | 2.56 | 1.07-6.15 | 0.036 | 0.616 |
|  | rs13243553 | 1.25 | 0.52-3.00 | 0.614 | 0.083 |
|  | rs2764261 | 1.75 | 0.72-4.23 | 0.214 | 0.052 |
|  | rs1248860 | 1.37 | 0.55-3.38 | 0.497 | 0.054 |
| IS |  |  |  |  |  |
|  | rs328902 | 1.09 | 0.36-3.27 | 0.881 | 0.554 |
|  | rs3781411 | 1.23 | 0.41-3.74 | 0.713 | 0.709 |
|  | rs13243553 | 1.09 | 0.36-3.31 | 0.886 | 0.546 |
|  | rs2764261 | 0.76 | 0.25-2.36 | 0.638 | 0.683 |
|  | rs1248860 | 0.76 | 0.24-2.40 | 0.644 | 0.660 |
| CS |  |  |  |  |  |
|  | rs328902 | 0.36 | 0.04-2.91 | 0.338 | 0.977 |
|  | rs3781411 | 1.43 | 0.18-11.64 | 0.739 | 0.134 |
|  | rs13243553 | 1.21 | 0.15-10.03 | 0.858 | 0.115 |
|  | rs2764261 | 1.23 | 0.15-10.33 | 0.851 | 0.115 |
|  | rs1248860 | 1.81 | 0.20-16.23 | 0.594 | 0.166 |
| SVS |  |  |  |  |  |
|  | rs328902 | 5.35 | 0.42-68.38 | 0.197 | 0.385 |
|  | rs3781411 | 7.54 | 0.58-98.05 | 0.123 | 0.545 |
|  | rs13243553 | 3.23 | 0.25-42.43 | 0.373 | 0.382 |
|  | rs2764261 | 1.63 | 0.12-22.22 | 0.712 | 0.815 |
|  | rs1248860 | 5.74 | 0.40-81.36 | 0.197 | 0.389 |
| LAS |  |  |  |  |  |
|  | rs328902 | 2.38 | 0.15-36.93 | 0.535 | 0.133 |
|  | rs3781411 | 5.76 | 0.37-90.97 | 0.213 | 0.087 |
|  | rs13243553 | 13.52 | 0.84-217.62 | 0.066 | 0.301 |
|  | rs2764261 | 7.64 | 0.46-126.79 | 0.156 | 0.107 |
|  | rs1248860 | 1.23 | 0.07-21.02 | 0.888 | 0.346 |

OR, odds ratio.CI confidence intervals. SNPs, single-nucleotide polymorphisms. AF, atrial fibrillation. CAD, coronary artery disease. MI, myocardial infarction. HF,heart failure. IS, ischemic stroke. LAS, large artery stroke. CS, cardioembolic stroke. SVS, Small vessel stroke.

**Supplementary Table 9.**Inverse variance weighted estimates for overall acceleration average and cardiovascular diseases, with SNPs individually removed in leave-one-out analyses

| Outcome | Excluded SNP | OR | 95%CI | P-value | P-value for heterogeneity |
| --- | --- | --- | --- | --- | --- |
| CAD |  |  |  |  |  |
|  | rs34517439 | 1.04 | 1.00-1.07 | 0.046 | 0.059 |
|  | rs6775319 | 1.03 | 0.97-1.09 | 0.314 | 0.009 |
|  | rs12522261 | 1.03 | 0.97-1.09 | 0.350 | 0.007 |
|  | rs9293503 | 1.01 | 0.96-1.07 | 0.724 | 0.022 |
|  | rs11012732 | 1.03 | 0.97-1.09 | 0.367 | 0.007 |
|  | rs148193266 | 1.02 | 0.96-1.08 | 0.553 | 0.007 |
|  | rs59499656 | 1.03 | 0.97-1.09 | 0.388 | 0.006 |
|  | rs113871181 | 1.01 | 0.97-1.04 | 0.730 | 0.082 |
| MI |  |  |  |  |  |
|  | rs34517439 | 1.03 | 0.99-1.07 | 0.155 | 0.064 |
|  | rs6775319 | 1.02 | 0.95-1.09 | 0.559 | 0.008 |
|  | rs12522261 | 1.02 | 0.95-1.09 | 0.605 | 0.007 |
|  | rs9293503 | 1.00 | 0.94-1.07 | 0.912 | 0.014 |
|  | rs11012732 | 1.02 | 0.95-1.09 | 0.604 | 0.007 |
|  | rs148193266 | 1.01 | 0.95-1.08 | 0.714 | 0.007 |
|  | rs59499656 | 1.02 | 0.96-1.09 | 0.458 | 0.012 |
|  | rs113871181 | 0.99 | 0.96-1.03 | 0.705 | 0.213 |
| AF |  |  |  |  |  |
|  | rs34517439 | 1.02 | 0.98-1.07 | 0.296 | 0.004 |
|  | rs6775319 | 1.02 | 0.96-1.07 | 0.571 | <0.001 |
|  | rs12522261 | 1.02 | 0.97-1.08 | 0.467 | <0.001 |
|  | rs9293503 | 1.00 | 0.95-1.06 | 0.855 | <0.001 |
|  | rs11012732 | 1.02 | 0.96-1.07 | 0.598 | <0.001 |
|  | rs148193266 | 1.01 | 0.96-1.07 | 0.644 | <0.001 |
|  | rs59499656 | 1.01 | 0.96-1.07 | 0.619 | <0.001 |
|  | rs113871181 | 0.99 | 0.96-1.02 | 0.499 | 0.106 |
| HF |  |  |  |  |  |
|  | rs34517439 | 0.98 | 0.96-1.01 | 0.283 | 0.083 |
|  | rs6775319 | 0.98 | 0.95-1.01 | 0.122 | 0.060 |
|  | rs12522261 | 0.97 | 0.95-1.00 | 0.051 | 0.136 |
|  | rs9293503 | 0.98 | 0.95-1.00 | 0.104 | 0.065 |
|  | rs11012732 | 0.98 | 0.96-1.01 | 0.236 | 0.065 |
|  | rs148193266 | 0.98 | 0.95-1.01 | 0.168 | 0.057 |
|  | rs59499656 | 0.99 | 0.97-1.02 | 0.663 | 0.459 |
|  | rs113871181 | 0.97 | 0.94-1.00 | 0.040 | 0.164 |
| IS |  |  |  |  |  |
|  | rs34517439 | 1.00 | 0.94-1.06 | 0.967 | 0.005 |
|  | rs6775319 | 1.00 | 0.94-1.07 | 0.991 | 0.006 |
|  | rs12522261 | 1.00 | 0.95-1.06 | 0.898 | 0.014 |
|  | rs9293503 | 0.97 | 0.92-1.03 | 0.342 | 0.020 |
|  | rs11012732 | 0.98 | 0.92-1.04 | 0.462 | 0.006 |
|  | rs148193266 | 0.99 | 0.92-1.06 | 0.696 | 0.002 |
|  | rs59499656 | 0.99 | 0.93-1.06 | 0.859 | 0.002 |
|  | rs113871181 | 0.98 | 0.92-1.04 | 0.515 | 0.004 |
| CS |  |  |  |  |  |
|  | rs34517439 | 0.98 | 0.91-1.05 | 0.560 | 0.481 |
|  | rs6775319 | 0.98 | 0.91-1.05 | 0.557 | 0.482 |
|  | rs12522261 | 0.99 | 0.93-1.06 | 0.857 | 0.615 |
|  | rs9293503 | 0.97 | 0.91-1.04 | 0.412 | 0.562 |
|  | rs11012732 | 0.96 | 0.90-1.03 | 0.305 | 0.714 |
|  | rs148193266 | 0.98 | 0.92-1.05 | 0.587 | 0.480 |
|  | rs59499656 | 1.00 | 0.94-1.08 | 0.921 | 0.836 |
|  | rs113871181 | 0.98 | 0.91-1.05 | 0.519 | 0.491 |
| SVS |  |  |  |  |  |
|  | rs34517439 | 0.98 | 0.83-1.16 | 0.819 | <0.001 |
|  | rs6775319 | 0.99 | 0.84-1.16 | 0.888 | 0.001 |
|  | rs12522261 | 0.98 | 0.83-1.16 | 0.818 | <0.001 |
|  | rs9293503 | 0.96 | 0.81-1.14 | 0.643 | <0.001 |
|  | rs11012732 | 0.92 | 0.79-1.07 | 0.285 | 0.002 |
|  | rs148193266 | 0.97 | 0.81-1.15 | 0.698 | <0.001 |
|  | rs59499656 | 0.97 | 0.81-1.15 | 0.716 | <0.001 |
|  | rs113871181 | 0.90 | 0.79-1.02 | 0.103 | 0.033 |
| LAS |  |  |  |  |  |
|  | rs34517439 | 0.95 | 0.87-1.03 | 0.215 | 0.871 |
|  | rs6775319 | 0.96 | 0.87-1.05 | 0.323 | 0.942 |
|  | rs12522261 | 0.94 | 0.86-1.03 | 0.186 | 0.865 |
|  | rs9293503 | 0.95 | 0.87-1.04 | 0.297 | 0.916 |
|  | rs11012732 | 0.93 | 0.85-1.02 | 0.111 | 0.932 |
|  | rs148193266 | 0.95 | 0.87-1.03 | 0.207 | 0.868 |
|  | rs59499656 | 0.94 | 0.86-1.03 | 0.187 | 0.866 |
|  | rs113871181 | 0.93 | 0.85-1.01 | 0.102 | 0.948 |

OR, odds ratio.CI confidence intervals. SNPs, single-nucleotide polymorphisms. AF, atrial fibrillation. CAD, coronary artery disease. MI, myocardial infarction. HF,heart failure. IS, ischemic stroke. LAS, large artery stroke. CS, cardioembolic stroke. SVS, Small vessel stroke.

**Supplementary Table 10.** Inverse variance weighted estimates for fraction of accelerations > 425 milli-gravities and cardiovascular diseases, with SNPs individually removed in leave-one-out analyses

| Outcome | Excluded SNP | OR | 95%CI | P-value | P-value for heterogeneity |
| --- | --- | --- | --- | --- | --- |
| CAD |  |  |  |  |  |
|  | rs1668835 | 0.84 | 0.46-1.53 | 0.573 | 0.002 |
|  | rs80028338 | 0.77 | 0.48-1.24 | 0.288 | 0.033 |
|  | rs743580 | 1.02 | 0.57-1.81 | 0.955 | 0.006 |
|  | rs4754194 | 1.00 | 0.57-1.75 | 0.988 | 0.006 |
|  | rs72633364 | 0.97 | 0.55-1.71 | 0.919 | 0.004 |
|  | rs62443625 | 0.89 | 0.48-1.64 | 0.710 | 0.001 |
|  | rs6433478 | 0.89 | 0.48-1.65 | 0.713 | 0.001 |
|  | rs1856329 | 0.76 | 0.44-1.31 | 0.329 | 0.011 |
| MI |  |  |  |  |  |
|  | rs1668835 | 0.83 | 0.45-1.51 | 0.539 | 0.007 |
|  | rs80028338 | 0.74 | 0.52-1.05 | 0.095 | 0.148 |
|  | rs743580 | 1.01 | 0.56-1.81 | 0.981 | 0.016 |
|  | rs4754194 | 0.95 | 0.52-1.75 | 0.880 | 0.008 |
|  | rs72633364 | 0.97 | 0.54-1.72 | 0.906 | 0.012 |
|  | rs62443625 | 0.87 | 0.47-1.63 | 0.670 | 0.004 |
|  | rs6433478 | 0.94 | 0.50-1.74 | 0.834 | 0.006 |
|  | rs1856329 | 0.78 | 0.43-1.41 | 0.413 | 0.013 |
| AF |  |  |  |  |  |
|  | rs1668835 | 1.36 | 0.87-2.15 | 0.181 | 0.002 |
|  | rs80028338 | 1.18 | 0.92-1.50 | 0.186 | 0.074 |
|  | rs743580 | 1.39 | 0.87-2.22 | 0.165 | 0.002 |
|  | rs4754194 | 1.46 | 0.97-2.21 | 0.070 | 0.009 |
|  | rs72633364 | 1.42 | 0.92-2.18 | 0.111 | 0.004 |
|  | rs62443625 | 1.38 | 0.88-2.17 | 0.165 | 0.002 |
|  | rs6433478 | 1.19 | 0.80-1.78 | 0.385 | 0.015 |
|  | rs1856329 | 1.31 | 0.83-2.09 | 0.246 | 0.001 |
| HF |  |  |  |  |  |
|  | rs1668835 | 1.06 | 0.82-1.38 | 0.650 | 0.419 |
|  | rs80028338 | 0.98 | 0.75-1.27 | 0.853 | 0.596 |
|  | rs743580 | 1.12 | 0.86-1.46 | 0.406 | 0.647 |
|  | rs4754194 | 1.04 | 0.80-1.35 | 0.760 | 0.385 |
|  | rs72633364 | 1.03 | 0.79-1.33 | 0.851 | 0.387 |
|  | rs62443625 | 1.07 | 0.82-1.39 | 0.620 | 0.436 |
|  | rs6433478 | 1.04 | 0.80-1.35 | 0.784 | 0.383 |
|  | rs1856329 | 0.96 | 0.74-1.25 | 0.755 | 0.696 |
| IS |  |  |  |  |  |
|  | rs1668835 | 0.77 | 0.55-1.07 | 0.124 | 0.205 |
|  | rs80028338 | 0.77 | 0.55-1.07 | 0.123 | 0.212 |
|  | rs743580 | 0.75 | 0.53-1.06 | 0.105 | 0.225 |
|  | rs4754194 | 0.92 | 0.66-1.29 | 0.648 | 0.420 |
|  | rs72633364 | 0.90 | 0.64-1.26 | 0.539 | 0.281 |
|  | rs62443625 | 0.85 | 0.61-1.19 | 0.335 | 0.139 |
|  | rs6433478 | 0.83 | 0.59-1.16 | 0.273 | 0.129 |
| CS |  |  |  |  |  |
|  | rs1856329 | 0.81 | 0.58-1.14 | 0.233 | 0.131 |
|  | rs1668835 | 0.63 | 0.33-1.20 | 0.158 | 0.759 |
|  | rs80028338 | 0.58 | 0.31-1.11 | 0.102 | 0.809 |
|  | rs743580 | 0.66 | 0.34-1.28 | 0.218 | 0.779 |
|  | rs4754194 | 0.68 | 0.35-1.29 | 0.235 | 0.819 |
|  | rs72633364 | 0.69 | 0.36-1.32 | 0.266 | 0.870 |
|  | rs62443625 | 0.59 | 0.31-1.12 | 0.106 | 0.800 |
|  | rs6433478 | 0.53 | 0.28-1.03 | 0.063 | 0.928 |
| SVS |  |  |  |  |  |
|  | rs1856329 | 0.65 | 0.34-1.25 | 0.201 | 0.779 |
|  | rs1668835 | 1.19 | 0.55-2.60 | 0.656 | 0.083 |
|  | rs80028338 | 0.82 | 0.37-1.80 | 0.619 | 0.871 |
|  | rs743580 | 1.38 | 0.62-3.06 | 0.432 | 0.089 |
|  | rs4754194 | 1.50 | 0.68-3.28 | 0.315 | 0.137 |
|  | rs72633364 | 1.37 | 0.62-2.99 | 0.435 | 0.090 |
|  | rs62443625 | 1.38 | 0.63-3.03 | 0.422 | 0.092 |
|  | rs6433478 | 1.42 | 0.64-3.15 | 0.390 | 0.098 |
| LAS |  |  |  |  |  |
|  | rs1856329 | 1.14 | 0.52-2.50 | 0.748 | 0.092 |
|  | rs1668835 | 0.61 | 0.26-1.41 | 0.246 | 0.810 |
|  | rs80028338 | 0.58 | 0.25-1.33 | 0.196 | 0.856 |
|  | rs743580 | 0.61 | 0.26-1.44 | 0.259 | 0.806 |
|  | rs4754194 | 0.61 | 0.26-1.41 | 0.249 | 0.809 |
|  | rs72633364 | 0.59 | 0.26-1.35 | 0.212 | 0.837 |
|  | rs62443625 | 0.64 | 0.28-1.47 | 0.290 | 0.800 |
|  | rs6433478 | 0.67 | 0.29-1.56 | 0.349 | 0.812 |
|  | rs1856329 | 0.81 | 0.35-1.87 | 0.618 | 0.996 |

OR, odds ratio.CI confidence intervals. SNPs, single-nucleotide polymorphisms. AF, atrial fibrillation. CAD, coronary artery disease. MI, myocardial infarction. HF,heart failure. IS, ischemic stroke. LAS, large artery stroke. CS, cardioembolic stroke. SVS, Small vessel stroke.

**Supplementary Table 11.**Mendelian Randomization estimates between overall acceleration average and cardiovascular diseases with MR-PRESSO outlier removed

| Outcome | Method | OR | 95% CI | P-value | SNPs |
| --- | --- | --- | --- | --- | --- |
| Atrial fibrillation | IVW | 1.00 | 0.97-1.03 | 0.864 | 6 |
|  | MR Egger | 1.09 | 0.96-1.22 | 0.182 | 6 |
|  | Weight median | 1.00 | 0.96-1.03 | 0.899 | 6 |
|  | MR-PRESSO | 1.00 | 0.97-1.03 | 0.871 | 6 |
| Coronary artery disease | IVW | 1.01 | 0.97-1.04 | 0.730 | 7 |
|  | MR Egger | 1.14 | 0.94-1.38 | 0.196 | 7 |
|  | Weighted median | 0.99 | 0.95-1.04 | 0.739 | 7 |
|  | MR-PRESSO | 1.01 | 0.96-1.06 | 0.809 | 7 |
| Myocardial infarction | IVW | 0.99 | 1.03-0.96 | 0.705 | 7 |
|  | MR Egger | 1.08 | 0.88-1.32 | 0.477 | 7 |
|  | Weighted median | 0.99 | 0.94-1.05 | 0.790 | 7 |
|  | MR-PRESSO | 0.99 | 0.95-1.04 | 0.759 | 7 |
| Ischemic stroke | IVW^§^ | 0.88 | 0.47-1.65 | 0.698 | 7 |
|  | MR Egger | 0.61 | 0-422087.07 | 0.943 | 7 |
|  | Weighted median | 0.61 | 0.26-1.44 | 0.258 | 7 |
|  | MR-PRESSO | 0.97 | 0.92-1.03 | 0.378 | 7 |
| Small vessel stroke | IVW^§^ | 0.90 | 0.79-1.02 | 0.103 | 7 |
|  | MR Egger | 0.86 | 0.48-1.56 | 0.622 | 7 |
|  | Weighted median | 0.89 | 0.79-1 | 0.053 | 7 |
|  | MR-PRESSO | 0.90 | 0.79-1.02 | 0.154 | 7 |

OR, odds ratio. CI, confidence intervals; IVW, inverse-variance-weighted method; MR-PRESSO, MR pleiotropy residual sum and outlier method. SNPs, single-nucleotide polymorphisms.Detailed information of outliers were shown in Supplemental Table 6.

^§^The estimates were evaluated from a random-effects IVW method due to the presence of heterogeneity based on Cochran’s Q.

**Supplementary Table 12.**Mendelian Randomization estimates between fraction of accelerations >425 milli-gravities and cardiovascular diseases with MR-PRESSOoutlier removed

| Outcome | Method | OR | 95% CI | P-value | SNPs |
| --- | --- | --- | --- | --- | --- |
| Atrial fibrillation | IVW | 1.18 | 0.92-1.5 | 0.186 | 7 |
|  | MR Egger | 0.56 | 0.00-494.04 | 0.866 | 7 |
|  | Weighted median | 1.08 | 0.77-1.52 | 0.647 | 7 |
|  | MR-presso | 1.18 | 0.84-1.64 | 0.376 | 7 |
| Coronary artery disease | IVW^§^ | 0.77 | 0.48-1.24 | 0.288 | 7 |
|  | MR Egger | 16.94 | 0-182893.82 | 0.550 | 7 |
|  | Weighted median | 0.82 | 0.5-1.36 | 0.449 | 7 |
|  | MR-presso | 0.77 | 0.48-1.24 | 0.329 | 7 |
| Myocardial infarction | IVW | 0.68 | 0.38-1.24 | 0.213 | 7 |
|  | MR Egger | 11.79 | 0-887584.36 | 0.667 | 7 |
|  | Weighted median | 0.72 | 0.34-1.55 | 0.407 | 7 |
|  | MR-presso | 0.74 | 0.48-1.15 | 0.232 | 7 |

OR, odds ratio. CI, confidence intervals; IVW, inverse-variance-weighted method; MR-PRESSO, MR pleiotropy residual sum and outlier method. SNPs, single-nucleotide polymorphisms.Detailed information of outliers were shown in Supplemental Table 6.

^§^The estimates were evaluated from a random-effects IVW method due to the presence of heterogeneity based on Cochran’s Q.
